# *d*-, *l*- and *d,l*-Tryptophan-Based Polyamidoamino Acids: pH-Dependent Structuring and Fluorescent Properties

**DOI:** 10.3390/polym11030543

**Published:** 2019-03-22

**Authors:** Federica Lazzari, Amedea Manfredi, Jenny Alongi, Daniele Marinotto, Paolo Ferruti, Elisabetta Ranucci

**Affiliations:** 1 Dipartimento di Chimica, Università degli Studi di Milano, via C. Golgi 19, 20133 Milano, Italy; amedea.manfredi@unimi.it (A.M.); jenny.alongi@unimi.it (J.A.); 2 Istituto di Scienze e Tecnologie Molecolari (ISTM-CNR), via C. Golgi 19, 20133 Milano, Italy; daniele.marinotto@istm.cnr.it

**Keywords:** polyamidoamino acid, *l*-tryptophan, pH-dependent solubility, pH-dependent circular dichroism, self-structuring, pH-dependent fluorescent properties

## Abstract

Chiral polyamidoamino acids were obtained by polyaddition of *N,N’*-methylenebisacrylamide with *d*-, *d*,*l*- and *l*-tryptophan (M-*d*-Trp, M-*d,l*-Trp and M-*l*-Trp). *l*-tryptophan/glycine copolymers, M-G-*l*-Trp_5_, M-G-*l*-Trp_10_, M-G-*l*-Trp_20_ and M-G-*l*-Trp_40,_ were prepared from *l*-tryptophan/glycine mixtures. These polymers were amphoteric, with acid-base properties similar to those of the parent amino acids. The *l*-tryptophan/glycine copolymers with high glycine content were water soluble in the pH range 2-12. M-G-*l*-Trp_40_ showed a solubility gap centred at pH 4.5 and all tryptophan homopolymers were soluble only at pH > 7. Dynamic light scattering measurements performed in their solubility ranges, namely 2-11 M-G-*l*-Trp_5_, M-G-*l*-Trp_10_ and M-G-*l*-Trp_20_ and 7-11 for M-G-*l*-Trp_40_, M-*d*-Trp, M-*l*-Trp and M-*d,l*-Trp, showed that the size of all samples did not significantly vary with pH. Both M-*l*-Trp and M-G-*l*-Trp copolymers showed pH-dependent circular dichroism spectra in the wavelength interval 200–280 nm, revealing structuring. All samples were fluorescent. Their emission spectra were unstructured and, if normalized for their tryptophan content, almost superimposable at the same pH, providing evidence that only tryptophan governed the photoluminescence properties. Changing pH induced in all cases a slight shift of the emission wavelength maximum ascribed to the modification of the microenvironment surrounding the indole ring induced by different protonation degrees.

## 1. Introduction

Bioinspired side-chain chiral homo- and copolymers derived from α-amino acids have received much attention in recent decades due to their potential as stimuli-responsive materials [1] with tunable pH- [2] and thermoreversible [3] solubility, chirality-dependent self-assembling via non-covalent forces [4,5] and chiral recognition [6] properties. Side-chain tryptophan-substituted polymers are of further interest thanks to their inherent fluorescence, which imparts them with potential for tracking in biological systems and in cellular and molecular imaging [7]. It is well known that the excitation of *l*-tryptophan residues is responsible for the fluorescence of proteins, frequently used as a diagnostic tool to explore their conformation [8]. Moreover, it has been demonstrated that *l*-tryptophan-rich peptides play a significant role on the cellular uptake and membrane interaction of arginine-rich cell penetrating peptides [9]. Whereas the hydrophobicity of the indole side substituent undoubtedly plays a role in that, it may be noticed that the hydrophobic moieties of other amino acids, such as for instance *l*-phenylalanine [9], were much less effective in this respect. Many examples of tryptophan-based polymers have been reported. In particular, poly-*N*-acryloyltryptophan was used in the determination of chiral interactions with 1,1-bis-2-naphthol [10]. Copolymers of *N*-acryloyltryptophan with other acrylamides [11] were employed in recognition studies [12,13,14]. The interaction of copolymeric poly-*N*-methacryloyltryptophan with β-cyclodextrin was investigated [15]. The tryptophan ester of polyhydroxyethylmethacrylate was also prepared, and its pH-dependent chiro-optical and fluorescence properties studied [16].

The Michael-type stepwise polyaddition of *prim*-amines or bis-*sec*-amines with bisacrylamides leads to linear polymers called polyamidoamines (PAAs) [17,18]. This reaction is specific, and in most cases, occurs under mild conditions. However, natural α-amino acids other than glycine were long considered unsuitable as monomers for polyaddition with bisacrylamides because they react very sluggishly under the usual PAA preparation conditions [19,20]. Subsequently, however, by changing the reaction conditions, the polyaddition of bisacrylamides with several natural α-amino acids was performed and led to a new family of PAA-related polymers named polyamidoamino acids (PAACs) [21,22]. The first PAAC, called *l*-ARGO7, used *l*-arginine and *N,N’*-methylenebisacrylamide (MBA) as monomers [20]. Subsequently, *d*- and *d*,*l-*ARGO7 were prepared from the corresponding arginine stereoisomers. *d*- and *l*-ARGO7 aqueous solutions yielded circular dichroism (CD) spectra showing molar ellipticity curves with pH-dependent maxima centered at 228 nm, suggesting the formation of stable conformations [21]. Theoretical modeling of *l*-ARGO7 gave evidence of a folded structure, with a slightly larger gyration radius for the largest chain positive charge at pH 1. Their main chain underwent transoid arrangements reminiscent of the protein hairpin motif. More recently, a small library of PAACs was similarly synthesized from *l*-alanine, *l*-valine and *l*-leucine and showed pH-dependent self-structuring in solution [23]. 

This paper reports on synthesis, acid-base properties, pH-dependent water solubility, structuring, chiro-optical and fluorescence properties of *l*-tryptophan/glycine copolymers with different *l*-tryptophan content, namely M-G-*l*-Trp_5_, M-G-*l*-Trp_10_, M-G-*l*-Trp_20_ and M-G-*l*-Trp_40_. Homopolymeric PAACs were also obtained by polyaddition of *N,N*’-methylenebisacrylamide with *d*-, *d*,*l*- and *l*-tryptophan, M-*d*-Trp, M-*d,l*-Trp and M-*l*-Trp, and their pH-dependent solubility in water assessed. 

## 2. Materials and Methods

**Materials.** Solvents and reagents, unless otherwise indicated, were analytical-grade commercial products and used as received. *d*-, *l*-, *d,l*-tryptophan (≥ 98%, 97% and 98% respectively) and glycine (≥ 99%) were purchased from Sigma-Aldrich (Milano, Italy). *N,N*’-Methylenebisacrylamide (MBA, 96%) purchased from Acros Organics (Milano, Italy) and LiOH monohydrate (≥ 98%) was supplied by Honeywell Fluka (Steinheim, Westphalia, Germany). HCl and NaOH volumetric standard solutions were purchased from Fluka analytics (Milano, Italy), while ethanol (≥ 98%) from Riedel-de-Haёn (Seelze, Hannover, Germany). Ultrapure water (18 MΩ·cm^−1^), produced with a Millipore Milli-Q^®^ apparatus (Darmstadt, Hesse, Germany), was used to prepare solutions.

**Characterizations.**^1^H and ^13^C NMR spectra were obtained in D_2_O at 25 °C using a Bruker Avance DPX-400 NMR operating (Bruker, Milano, Italy) at 400.13 MHz (d1 = 10 s) and 100.40 MHz, respectively. Prior to the analysis, polymers were dissolved in water and basified with 0.1 M NaOH until pH 10. The final product was freeze-dried and dissolved in D_2_O. 

Fourier-Transform Infrared spectroscopy in Attenuated Total Reflectance configuration (FTIR-ATR) spectra were recorded performing 16 scans at 4 cm^−1^ resolution in the 4000–500 cm^−1^ range, using a Perkin Elmer Spectrum 100 spectrometer (Milano, Italy) equipped with a diamond crystal (penetration depth = 1.66 μm). Before analysis, samples were dried under a vacuum to constant weight. 

Size exclusion chromatography (SEC) traces were obtained for all copolymers with Toso-Haas TSK-gel G4000 PW and TSK-gel G3000 PW columns connected in series, using a Waters model 515 HPLC pump (Milano, Italy) equipped with a Knauer autosampler 3800 (Knauer, Bologna, Italy), a light scattering (670 nm), a viscometer Viscotek 270 dual detector (Malvern, Roma, Italy) and a refractive index detector (Waters, Model 2410, Milano, Italy). The mobile phase was a 0.1 M Tris buffer (pH 8.00 ± 0.05) solution with 0.2 M sodium chloride. Sample concentration: 20 mg mL^−1^; flow rate: 1 mL min^−1^; injection volume: 20 µL; loop size: 20 µL; column dimensions: 300 × 7.5 mm^2^.

Dynamic light scattering (DLS) analyses were carried out on 1 mg mL^−1^ polymer solutions prepared in ultrapure water, using a Malvern Zetasizer NanoZS instrument (Malvern, Roma, Italy), equipped with a laser fitted at 532 nm and fixed 173° scattering angle. Before analyses, samples were filtered through a 0.2 µm syringe Whatman filter. The solution pH was adjusted to the selected value using 0.1 M HCl or 0.1 M NaOH aqueous solutions. Measurements were performed in triplicate, and each value was reported as the average of 10 runs.

Circular dichroism (CD) spectra were obtained using a JASCO J-500CD spectrometer (Jasco Europe Srl, Lecco, Italy), by scanning from 200 to 300 nm in a 1 cm path-length quartz cell at 50 nm min^−1^ scan speed. Each spectrum reported was the average of 3 measurements. 0.5 mg·mL^−1^ polymer solutions were prepared by dissolving polymer samples in 0.1 M NaCl solution. The pH was adjusted using 0.1 M HCl or 0.1 M NaOH aqueous solutions and measured by a combined Metrohm microelectrode (Varese, Italy). CD spectra were normalized based on the molar concentration of tryptophan-bearing repeat units, then reported as molar ellipticity (*θ* expressed as mdeg M^−1^·cm^−1^). 

The *pK*_a_ values were determined by potentiometric titration from the half-neutralization points, where pH = *pK*_a_, following the procedure described in the Supplementary Materials (Figure S1 and Table S1). For *a* ≠ 0.5, polyelectrolyte behavior is described by the modified Henderson-Hasselbalch equation (Equation (1)):

(1) $$pH=pK_{a}-\beta \;log\frac{1-\alpha }{\alpha }$$

where *K*_a_ is the apparent acidic dissociation constant of the group being pH-determining in the buffer titration zone considered and *β* is the Katchalsky and Spitnik parameter [24] accounting for possible interactions between ionizable groups of repeat units being spatially or topologically adjacent. *β* values (Table 3) were determined by following the procedures described in the Supplementary Materials (see also Figure S2).

Titrations were performed according to the following procedure. Samples were dissolved in a 0.1 M NaCl aqueous solution (10 mL) in order to obtain a 0.05 M repeating unit solution. Solutions, deaerated by continuous ultrapure N_2_ bubbling and thermostated at 25 °C, were pH-metrically forward titrated with 0.1 M NaOH. The solution pH was adjusted to 1.2-1.3 using 1 M HCl (0.7 mL). Due to solubility limits, M-G-*l*-Trp_40_ was back titrated with 0.1 M HCl starting from pH 12.3-12.4 adjusted with 0.1 M NaOH (0.7 mL). The pH-meter, a Primatrode with a NTC electrode connected to an 827 pH lab Metrohm, was calibrated against two pH standard buffers, thermostated at 25 °C. All titration experiments were performed in quadruplicate.

Solubility tests in aqueous media at different pH’s were determined by recording the transmittance at 450 nm using a Perkin-Elmer Lambda 35 spectrometer using plastic cuvettes with 1 cm path length. Solutions were prepared by dissolving 20 mg polymer in 0.1 M NaOH (2.7 mL), adjusting the pH with 0.1 M or 0.01 M HCl aqueous solutions, and finally diluting with ultrapure water to 1 mg·mL^−1^ concentration. The solutions were thermostated for 90 min at 30 °C before measurements. Measurements were performed in triplicate. The scattering of a polarized IR beam in ultrapure water at different pH’s was used to detect the presence of aggregates, if any.

Absolute photoluminescence quantum yield, Φ, was measured using a C11347 Quantaurus Hamamatsu Photonics K.K spectrometer (Hamamatsu City, Shizuoka, Japan), equipped with a 150 W Xenon lamp, an integrating sphere and a multichannel detector. Φ was calculated using Equation (2):

(2) $$\Phi =\;\frac{PN\left(em\right)}{PN\left(abs\right)}=\;\frac{\int \frac{\lambda }{hc}⎡I_{em}^{sample}\left(\lambda \right)-\;I_{em}^{reference}\left(\lambda \right)⎤d\lambda }{\int \frac{\lambda }{hc}⎡I_{exc}^{reference}\left(\lambda \right)-\;I_{exc}^{sample}\left(\lambda \right)⎤d\lambda }$$

where *PN(em)* is the number of emitted photons, *PN(abs)* the number of absorbed photons, *λ* the wavelength, *h* the Planck’s constant, *c* the speed of light, $I_{em}^{sample}$ and $I_{em}^{reference}$ the photoluminescence intensities of the sample solution in ultrapure water and of water, respectively, $I_{exc}^{sample}$ and $I_{exc}^{reference}$ the excitation light intensities of the sample solution in ultrapure water and of water, respectively. The error made was estimated to be around 5%.

Steady state and time-resolved fluorescence data were obtained using a FLS980 spectrofluorimeter (Edinburgh Instrument Ltd, Livingston, Scotland, UK). Emission spectra were recorded exciting at 279 nm, corrected for background intensity and quantum efficiency of the photomultiplier tube. Excitation spectra were carried out at the maximum of the emission spectrum and corrected for the intensity fluctuation of a 450 W Xenon arc lamp.

Time-resolved fluorescence measurements were performed through the time-correlated single photon counting technique with an Edinburgh Picosecond Pulsed Diode Laser EPLED-300 (Livingston, Scotland, UK): emitted wavelength 301 nm, temporal pulse width (FWHM) 857 ps. A Ludox solution was used as scatter to determine the instrument response function (IRF). Time-resolved fluorescence curves were reconvoluted using the IRF and a multi-exponential impulse response function (Equation (3)):

(3) $$I\left(\lambda ,t\right)=\;\overset{n}{\underset{i=1}{\sum }}\alpha_{i}\left(\lambda \right)\exp\left(\frac{-t}{\tau_{i}}\right)$$

where *n* is the number of exponentials, *α*_i_ (λ) is the amplitude at wavelength λ and *τ*_i_ is the lifetime of the component *i*. Quality of the fit was evaluated through the reduced $\chi^{2}$ values. Two different sets of 1 cm path length quartz cells were employed for the photoluminescence analysis: the first ones were classic fluorescent cuvettes for non-degassed solutions, the second ones have been built specifically to perform freeze-pump-thaw cycles to remove dissolved oxygen into the solution. In order to degas the solution as much as possible, three freeze-pump-thaw cycles were executed using a turbomolecular pump. 

Steady-state, time resolved and quantum yields measurements were carried out at room temperature on *l*-tryptophan, M-*l*-Trp and M-G-*l*-Trp_5_, M-G-*l*-Trp_10,_ M-G-*l*-Trp_20_ and M-G-*l*-Trp_40_ at pH 11, 7-8 and 1.5-2, considering the solubility limits. The pH was adjusted using 0.1 M HCl or 0.1 M NaOH aqueous solutions and measured by a combined Metrohm microelectrode. 

**Synthesis of l-tryptophan based homo- and copolymers*. M-l-Trp*.** Thermostated and deaerated ultrapure water (4 mL) was added to a mixture of *l*-tryptophan (3.54 g, 17.33 mmol) and MBA (2.79 g, 18.01 mmol) at 50 °C under magnetic stirring. After 5 min, a thermostated and deaerated LiOH monohydrate aqueous solution (0.36 g; 8.60 mmol; 2 mL) was added to the mixture. After 2 h, a second portion of LiOH solution (0.36 g; 8.60 mmol; 2 mL) was introduced. The reaction mixture was kept at 50 °C for 6 days under nitrogen atmosphere. After this time, the solution was acidified to pH 3.5 with 6 M HCl, inducing separation of crude M-*l*-Trp in form of a brown oily liquid. The product was extracted five times with ethanol (20 mL) until the formation of a brown powder. The polymer was further dried under vacuum until constant weight (yield: 92%). Subsequently, 2 g were solubilized in H_2_O at pH 9 and ultrafiltered through membranes with 100000 and then 5000 as nominal molecular weight cut-off. The solution passed through the former and retained by the latter was freeze-dried recovering the product as a yellowish powder. 

^1^H NMR (D_2_O, 400.132 MHz, ppm): *δ* 2.01–2.08 (m, 4H, COC**H_2_**CH_2_N) 2.33–2.93 (m, 6H, COCH_2_C**H_2_**N and C**H_2_**CHCOO^−^), 3.86 and 4.16–4.27 (m, 2H, NHC**H_2_**NH), 3.93–4.00 (m, 1H, CH_2_C**H**COO^−^), 5.68–5.71 and 6.13–6.15 ppm (m, 3H, **H_2_**C=C**H** of terminal acrylamide), 6.90–7.06 (m, 3H, **H_F_**, **H_G_**, **H_H_** of *l*-tryptophan), 7.21–7.52 (m, 2H, **H_I_**, **H_L_** of *l*-tryptophan) (Figure S3). ^13^C NMR (D_2_O, 100.623, ppm): *δ* 16.99, 26.10, 28.69, 33.42, 42.97, 43.73, 46.40, 57.40, 63.85, 64.86, 65.83, 110.79, 111.76, 118.51, 118.72, 119.03, 121.68, 123.65, 124.04, 127.05, 128.05, 136.14, 174.13, 179.54, 181.62. 

***M-d-Trp*** and ***M-d,l-Trp*** were prepared as M-*l*-Trp by substituting *d*-Trp and *d,l*-Trp for *l*-Trp. Yields 94% and 98%.

***M-G-l-Trp_5_*.** Thermostated and deaerated ultrapure water (5 mL) was added to a mixture of *l*-tryptophan (0.53 g, 2.60 mmol), glycine (3.59 g, 47.80 mmol) and MBA (8.09 g, 52.50 mmol) at 50 °C under magnetic stirring. After 5 min, thermostated and deaerated LiOH monohydrate solution (1.07 g, 25.50 mmol, 5 mL) was added to the mixture. After 2 h, a second portion of LiOH (0.36 g, 8.60 mmol, 2 mL) was introduced. The reaction mixture was maintained at 50 °C for 6 days under nitrogen atmosphere. After this time, the solution was acidified to pH 3.5 with 6 M HCl and ultrafiltered as in the case of M-*l*-Trp. Yield: 90 %. $M_{w}$ = 13,000; $M_{w}/M_{n}$ = 1.44 (Figure S4). 

^1^H NMR (D_2_O, 400.132 MHz, ppm): *δ* 2.19–2.22 (m, 4H, COC**H_2_**CH_2_N of *l*-tryptophan bearing units), 2.32 (t, 4H, COC**H_2_**CH_2_N of glycine bearing units), 2.61-2.62 (m, 4H, COCH_2_C**H_2_**N of *l*-tryptophan bearing units), 2.73–2.76 (m, 4H, COCH_2_C**H_2_**N of glycine bearing units), 2.93 (s, 2H, C**H_2_**CHCOO^−^ of *l*-tryptophan), 3.04 (s, 2H, C**H**COO^−^ of glycine), 4.29 (s, 1H, C**H**COO^−^ of *l*-tryptophan), 4.35 (s, 1H, NHC**H_2_**NH of *l*-tryptophan bearing units), 4.44–4.47 (m, 3H, NHC**H_2_**NH of both *l*-tryptophan and glycine bearing units), 4.56 (s, 2H, NHC**H_2_**NH of terminal acrylamide), 5.68–5.71 and 6.13–6.15 ppm (m, 3H, **H_2_**C=C**H** of terminal acrylamide), 7.04–7.13 (m, 3H, **H_G_**, **H_H_**, **H_I_** of *l*-tryptophan), 7.36–7.40 (m, 1H, **H_L_** of *l*-tryptophan), 7.60–7.62 (m, 1H, **H_M_** of *l*-tryptophan) (Figure S3). ^13^C NMR (D_2_O, 100.623, ppm): *δ* 32.87, 44.34, 49.33, 57.33, 175.32, 178.83. Content of *l*-tryptophan-bearing units by ^1^H NMR = 4.53%.

***M-G-l-Trp_10_*** was prepared as M-G-*l*-Trp_5_ using a different *l*-tryptophan/glycine ratio (1.04 g, 5.10 mmol for *l*-tryptophan and 3.41 g, 45.40 mmol for glycine). Yield 72%. $M_{w}$ = 11,400; $M_{w}/M_{n}$ = 1.30 (Figure S4). 

^1^H NMR (D_2_O, 400.132 MHz, ppm): *δ* 2.19–2.22 (m, 4H, COC**H_2_**CH_2_N of *l*-tryptophan bearing units), 2.31–2.34 (m, 4H, COC**H_2_**CH_2_N of glycine bearing units), 2.61–2.62 (m, 4H, COCH_2_C**H_2_**N of *l*-tryptophan bearing units), 2.73-2.76 (m, 4H, COCH_2_C**H_2_**N of glycine bearing units), 2.93 (s, 3H, C**H_2_**CHCOO^−^), 3.04 (s, 2H, C**H**COO^−^ of glycine), 4.29 (s, 1H, C**H**COO^−^ of *l*-tryptophan), 4.35 (s, 1H, NHC**H_2_**NH of *l*-tryptophan bearing units), 4.44–4.47 (s, 2H, NHC**H_2_**NH of both *l*-tryptophan and glycine bearing units), 4.56 (s, 2H, NHC**H_2_**NH of terminal acrylamide), 5.68–5.70 and 6.13–6.15 ppm (m, 3H, **H_2_**C=C**H** of terminal acrylamide), 7.05–7.13 (m, 3H, **H_G_**, **H_H_**, **H_I_** of *l*-tryptophan), 7.36–7.38 (m, 1H, **H_L_** of *l*-tryptophan), 7.54–7.62 (m, 1H, **H_M_** of *l*-tryptophan) (Figure S3). ^13^C NMR (D_2_O, 100.623, ppm): *δ* 32.85, 44.32, 49.30, 51.94, 57.32, 118.52, 121.68, 123.67, 127.03, 136.14, 168.44, 175.21, 178.81. Content of *l*-tryptophan bearing units from ^1^H NMR = 9.70%.

***M-G-l-Trp_20_*** was prepared as M-G-*l*-Trp_5_ using a different *l*-tryptophan/glycine ratio (2.08 g, 10.20 mmol for *l*-tryptophan and 3.03 g, 40.40 mmol for glycine). Yield 72%. $M_{w}$ = 11,200; $M_{w}/M_{n}$ = 1.30 (Figure S4). 

^1^H NMR (D_2_O, 400.132 MHz, ppm): *δ* 2.19–2.22 (m, 4H, COC**H_2_**CH_2_N of *l*-tryptophan bearing units), 2.30–2.34 (m, 4H, COC**H_2_**CH_2_N of glycine bearing units), 2.59–2.62 (m, 4H, COCH_2_C**H_2_**N of *l*-tryptophan bearing units), 2.72–2.74 (m, 4H, COCH_2_C**H_2_**N of glycine bearing units), 2.93 (s, 3H, C**H_2_**CHCOO^−^), 3.04 (s, 2H, C**H**COO^−^ of glycine), 4.29 (s, 1H, C**H**COO^−^ of *l*-tryptophan), 4.35 (s, 1H, NHC**H_2_**NH of *l*-tryptophan bearing units), 4.44–4.47 (s, 2H, NHC**H_2_**NH of both *l*-tryptophan and glycine bearing units), 4.56 (s, 2H, NHC**H_2_**NH of terminal acrylamide), 5.67–5.70 and 6.12–6.15 ppm (m, 3H, **H_2_**C=C**H** of terminal acrylamide), 7.05–7.13 (m, 3H, **H_G_**, **H_H_**, **H_I_** of *l*-tryptophan), 7.36–7.38 (m, 1H, **H_L_** of *l*-tryptophan), 7.54–7.60 (m, 1H, **H_M_** of *l*-tryptophan) (Figure S3). ^13^C NMR (D_2_O, 100.623, ppm): *δ* 32.85, 44.32, 49.30, 51.94, 57.32, 118.52, 121.68, 123.67, 127.03, 136.14, 168.44, 175.21, 178.81. Content of *l*-tryptophan bearing units from ^1^H NMR from ^1^H NMR = 17.40%.

***M-G-l-Trp_40_*** was prepared as M-*l*-Trp by substituting an *l*-tryptophan/glycine mixture (*l*-tryptophan 34.17 g, 20.42 mmol; glycine 2.27 g, 30.24 mmol) for *l*-tryptophan. The product was extracted five times with EtOH (20 mL) until the formation of a brown powder, then dried under vacuum until constant weight (yield: 41%). $M_{w}$ = 20,300; $M_{w}/M_{n}$ = 2.45 (Figure S4).

^1^H NMR (D_2_O, 400.132 MHz, ppm): *δ* 2.11 (s, 4H, COC**H_2_**CH_2_N of *l*-tryptophan bearing units), 2.22 (s, 4H, COC**H_2_**CH_2_N of glycine bearing units), 2.51 (m, 4H, COCH_2_C**H_2_**N of *l*-tryptophan bearing units), 2.65 (m, 4H, COCH_2_C**H_2_**N of glycine bearing units), 2.86 (s, 2H, C**H_2_**CHCOO^−^), 2.96 (s, 2H, C**H**COO^−^ of glycine), 4.06–4.23 (m, 3H, C**H**COO^-^ and NHC**H_2_**NH of *l*-tryptophan bearing units), 4.39–4.47 (m, 3H, NHC**H_2_**NH of both *l*-tryptophan and glycine bearing units), 5.55–5.63 and 5.99–6.07 ppm (m, 3H, **H_2_**C=C**H** of terminal acrylamide), 6.98–7.02 (m, 3H, **H_G_**, **H_H_**, **H_I_** of *l*-tryptophan), 7.30 (m, 1H, **H_L_** of *l*-tryptophan), 7.45–7.55 (m, 1H, **H_M_** of *l*-tryptophan) (Figure S3). ^13^C NMR (D_2_O, 100.623, ppm): *δ* 32.67, 33.76, 35.08, 44.04, 46.52, 49.19, 57.36, 63.91, 65.78, 111.64, 118.59, 119.02, 121.68, 122.40, 123.99, 127.04, 136.13, 175.21, 178.74, 179.44, 181.65. Content of *l*-tryptophan bearing units from ^1^H NMR from ^1^H NMR = 40.5%.

## 3. Results and Discussion

### 3.1. Synthesis of M-l-Trp and M-G-l-Trp Copolymers

**Scheme 1:**
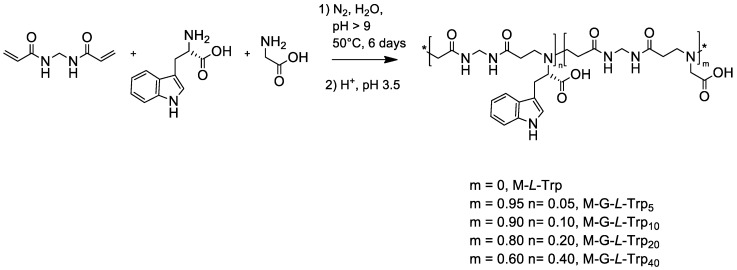
Synthesis of *l*-tryptophan based homo- and copolymers.

*d-*, *l*- and *d,l*-Tryptophan polyamidoamino acids were prepared in water at 50 °C and pH > 9 for 6 days by polyaddition of *l*-, *d*-, and *d,l*-tryptophan with *N,N*’-methylenebisacrylamide (MBA) as reported in Scheme 1 for the *l*-isomer. Glycine copolymers were prepared in the same way by partly substituting glycine for tryptophan in the preparation recipe.

The above synthetic procedure was similar to that previously described for MBA-arginine (ARGO7) [22], MBA-*l*-alanine (M-*l*-Ala), MBA-*l*-valine (M-*l*-Val) and MBA-*l*-leucine (M-*l*-Leu) [23], but with few differences. In all polyadditions of acid- or neutral α-amino acids with bisacrylamides, including the alanine, valine and leucine mentioned above, a molar equivalent of strong alkali per carboxyl group was added from the beginning to the reaction mixture in order to de-protonate the amine groups. In the case of tryptophan, however, additional precautions had to be adopted, because tryptophan is highly sensitive to O_2_-mediated oxidation in basic environment [25,26]. Therefore, in the present case, the reaction mixture was carefully flushed throughout with ultrapure nitrogen and, moreover, the best procedure involved the slow addition of the base to the reacting mixture. This resulted in a reacting mixture containing limited amounts of tryptophan sodium salt buffered by excess free tryptophan. Only the tryptophan amount salified at each base addition reacted. Noticeably, once the tryptophan amine groups had reacted with the MBA double bonds, no more blackening due to oxidation was observed. Probably, if the base is portion wise added to the reaction mixture, the resultant substituted amine groups, owing to the presence of carbonyl groups in β-position, were not basic enough to de-protonate the indole ring; hence, the amino acid *prim*-amine groups were stepwise activated by deprotonation at a rate roughly matching the rate of the addition reaction. 

**Figure 1:**
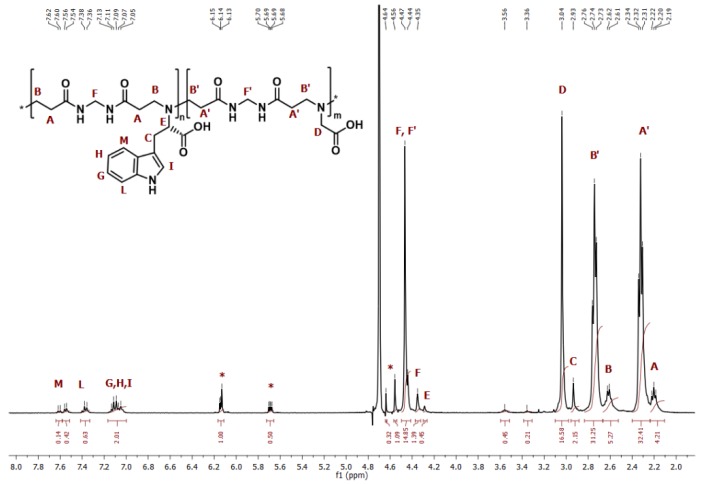
^1^H NMR spectrum of M-G-*l*-Trp_10_, recorded in D_2_O at 25 °C. Asterisks represent the signals of methylene and double bond protons of the terminal acrylamide.

Copolymeric *l*-tryptophan/glycine PAACs were similarly prepared from *l*-tryptophan/glycine mixtures with tryptophan content ranging from 5% to 40%, on a molar basis, as indicated by the subscripts of their acronyms, namely M-G-*l*-Trp_5_, M-G-*l*-Trp_10_, M-G-*l*-Trp_20_ and M-G-*l*-Trp_40_. The structure of homo- and copolymers was confirmed by ^1^H (Figure 1 and Figure S3), ^13^C NMR (see also Materials and Methods) and FTIR-ATR (Figure S5) analyses. It may be observed that, in the ^1^H NMR spectra, the peaks of terminal acrylamide groups are apparent and, moreover, those assigned to the tryptophan M hydrogen are split, possibly due to conformational effects. The molar ratios in the reaction recipes were not far from those found in the resultant copolymers (Table 1).

The borderline solubility in the mobile phase normally adopted in SEC analyses of PAAs and PAACs, that is, TRIS buffer pH 8 added with sodium chloride, discouraged the use of this technique for determining the molecular weight values of M-*d*-Trp, M-*l*-Trp and M-*d,l*-Trp. Therefore, their number-average molecular weights (*M*_n_) were estimated in the range 3500–5000 by end-group counting in their ^1^H NMR spectra recorded in D_2_O at pH 10 and calculated by considering the easily determined terminal double bonds. Two cases were considered: (1) both polymer terminals bear acrylamide double bonds; (2) polymers bear statistically only one acrylamide double bond terminal per macromolecule. The results were compared with those of SEC, when determined. In both cases, the trend was decreasing *M*_n_ by increasing tryptophan content (Table 2). However, the *M*_n_ values determined by SEC were in better agreement with those calculated from ^1^H NMR by supposing two acrylamide double bond terminals per macromolecule. 

Weight (*M*_w_) and number-average molecular weights (*M*_n_) were determined by SEC using right and small angle light scattering detectors equipped with a laser fitted at 670 nm. Interference from tryptophan fluorescence was not considered because the tryptophan excitation maximum is set at 280 nm [27]. 

### 3.2. Acid-Base Properties

The *pK*_a_ values of M-G-*l*-Trp copolymers (Table 3) were determined by potentiometric titration following the previously-reported procedure [28] as described in Materials and Methods and in Supplementary Materials (Figure S1 and Table S1). It may be observed that titration curves showed only two inflection points and two buffer regions corresponding to similar acid-base properties of glycine and *l-*tryptophan bearing units. This result is in line with the observation that the parent amino acids have almost the same *pK*_a_ values, respectively 2.34 (carboxyl group) and 9.6 (amine group) for glycine and 2.83 and 9.30 for tryptophan.

**Figure 2:**
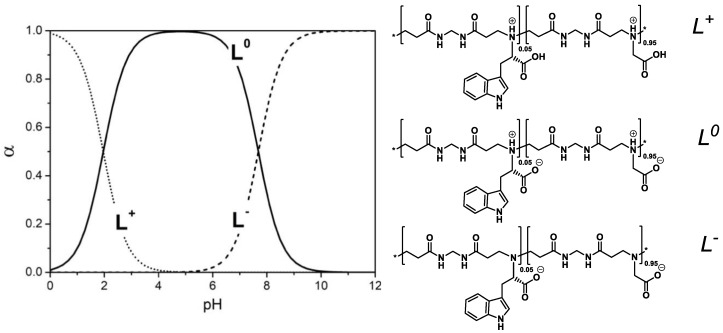
Speciation diagram and ionization states of M-G-*l*-Trp_5_.

Glycine and *l*-tryptophan repeat units in the M-G-*l*-Trp copolymers can exist in three ionization states. The pH-dependent speciation curves (reported in Figure 2 for M-G-*l*-Trp_5_) were determined from the *pK*_a_ and *β* values reported in Table 3 following methods described in the Supplementary Materials.

### 3.3. Solubility Properties

**Figure 3:**
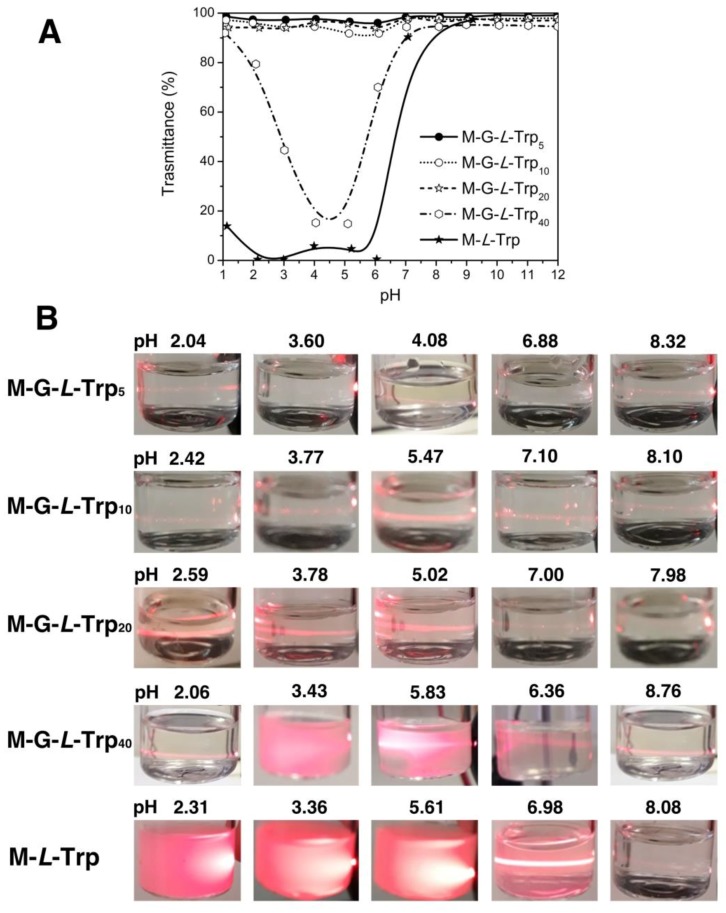
Solubility of M-G-*l*-Trp copolymers in water. (**A**) pH-dependence of transmittance at 480 nm; (**B**) scattering of a polarized IR beam. Measurements were performed on 1 mg·mL^−1^ solutions at 25 °C.

Tryptophan-based PAACs showed composition- and pH-dependent solubility, as ascertained by UV–Vis measurements (Figure 3A) and scattering of polarized IR beam (Figure 3B) tests carried out on 1 mg·mL^−1^ polymer solutions at different pHs. Light transmittance measurements were carried out at 480 nm, that is, outside PAACs’ absorption wavelength range. Copolymers with highest glycine content, namely M-G-*l*-Trp_5_, M-G-*l*-Trp_10_ and M-G-*l*-Trp_20_, showed complete solubility in the whole 2–12 pH range. M-G-*l*-Trp_40_ showed a solubility gap centered at pH 4.5, that is, very close to the polymer IP, whereas M-*l*-Trp proved soluble only at pH > 7. The polarized IR beam scattering confirmed these results. 

### 3.4. Dynamic Light Scattering (DLS) Measurements 

**Figure 4:**
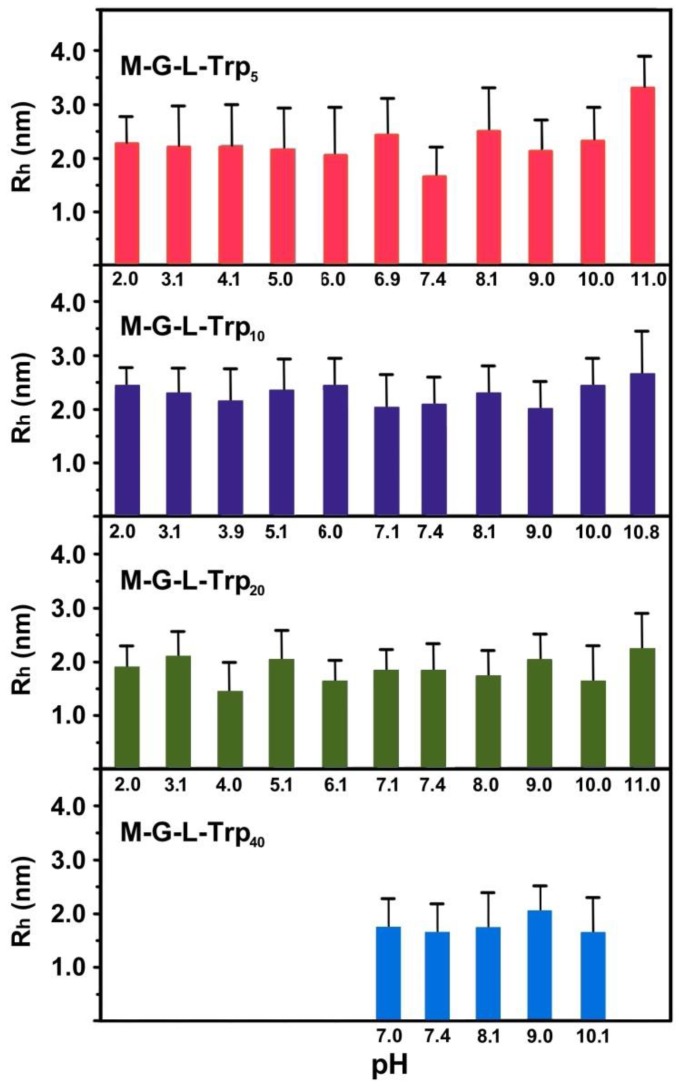
pH-dependence of the hydrodynamic radius of M-G-*l*-Trp copolymers. Data were obtained in 0.1 M NaCl at 1 mg·mL^−1^ and 25 °C.

The pH-dependence of the hydrodynamic radii, *R*_h_, of tryptophan-based PAACs was determined by DLS measurements at 1 mg·mL^−1^ in 0.1 M NaCl. Data were recorded within the pH range 2-11 for M-G-*l*-Trp_5_, M-G-*l*-Trp_10_ and M-G-*l*-Trp_20_, and within the pH range 7–11 for M-G-*l*-Trp_40_, M-*l*-Trp, M-*d,l*-Trp and M-*d*-Trp. Monomodal volumetric distributions were obtained in all cases. As already observed for other PAACs [23], the *R*_h_ values of all samples did not significantly vary within their solubility pH range (Figure 4). 

**Figure 5:**
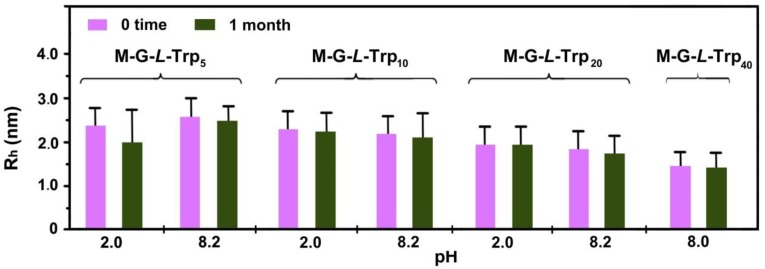
Hydrodynamic radius of M-G-*l*-Trp copolymers at 0 and 1 month. Data were recorded in 0.1 M NaCl at 1 mg·mL^−1^ and 25 °C.

The stability on time of the M-G-*l*-Trp copolymer solutions were tested after one month at pH 2 and 8 (Figure 5). *R*_h_ did not show significant variations.

**Figure 6:**
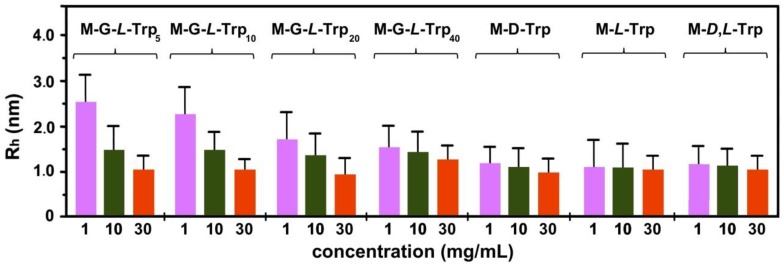
Hydrodynamic radius of M-G-*l*-Trp copolymers in the 1–30 mg·mL^−1^ range. Data were recorded in 0.1 M NaCl at pH 8 and 25 °C.

Homo- and copolymers behaved differently in terms of *R*_h_ dependence on concentration. By decreasing concentration, that of copolymers increased, suggesting a polyelectrolyte effect (Figure 6), that is, in dilute solutions charges were less shielded and coil expansion occurred. By contrast, the *R*_h_ of homopolymers was little affected by concentration variations in the 1–30 mg·mL^−1^ range, being overshadowed by a more rigid conformation induced by the superior bundling ability of the bulky, hydrophobic indole substituents.

### 3.5. Circular Dichroism Analysis

**Figure 7:**
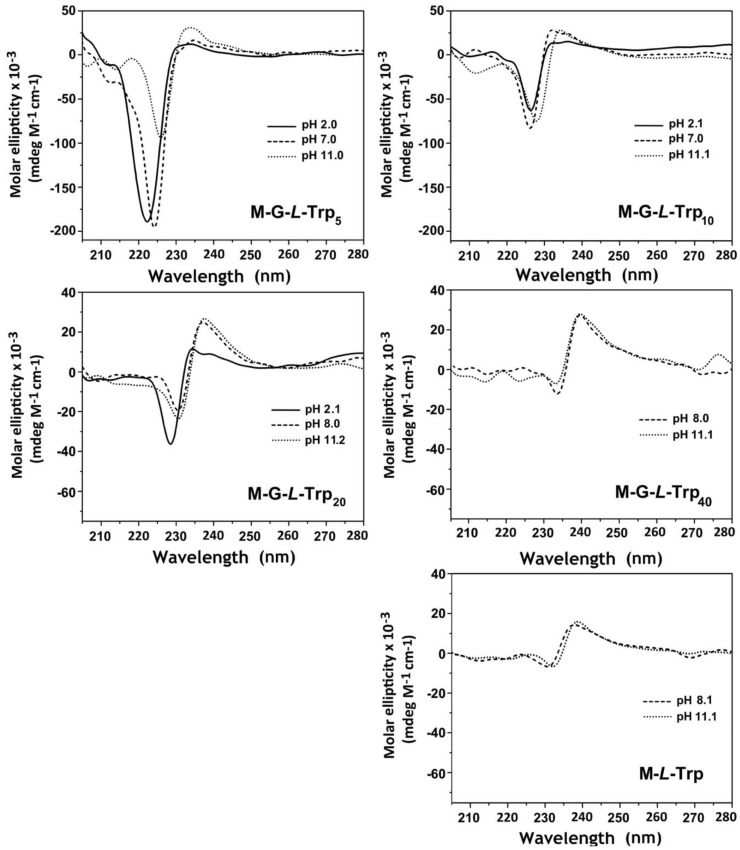
pH-Dependence of M-G-*l*-Trp CD spectra. Data were obtained in 0.1 M NaCl solution at 25 °C. The overall concentration of the repeating units was 1.81 ± 0.25 mM. The curves were normalized accounting for the molar concentration of tryptophan units.

The CD spectra of M-*l*-Trp and M-G-*l*-Trp in 0.1 M NaCl at pH 2, 7–8 and 11 are shown in Figure 7, where curves were normalized with respect to the molar concentration of the tryptophan units. These spectra demonstrated that all polymers self-assembled into stable conformations, whose behavior significantly depended on tryptophan content and pH. The pH-dependence may be considered a general feature of PAACs’ CD spectra, regardless of the nature of the amino acid residue, as shown by MBA-arginine (ARGO7) [22], bearing cationic residues, MBA-*l*-alanine (M-*l*-Ala), MBA-*l*-valine (M-*l*-Val) and MBA-*l*-leucine (M-*l*-Leu), bearing hydrophobic residues [23].

The CD spectrum of M-G-*l*-Trp_5_ was the most intense and highly affected by pH. At pH 2.0, the negative band centered at 222 nm was mainly ascribed to the weak n → π^*^ transition of the CONH groups [27]. Literature data show that at 220 nm the high-energy π → π^*^ B_b_ indole transition induces a positive band [29,30] that, here, was most probably masked by the CONH absorption. Such a high intensity was associated to a strong dipole moment, which was probably caused by specific conformations assumed by the sequences of glycine-bearing repeat units present in large excess. At pH 7.0, 22% of the main chain *tert*-amine groups were deprotonated and the average net charge per repeat unit was −0.14. This was probably causing the 2 nm shift visible in the CD spectra. At pH 11.0, all *tert*-amine groups were deprotonated, and the net charge per repeat unit was −1. This caused changes in the CD spectrum in both intensities and wavelength in which maxima were found. In particular, a decrease in the intensity of the negative peak was recorded, centered at 226 nm. A slight increase in the positive peak was also observed. Apparently, as for the other PAACs studied so far, the protonation degree of the main *tert*-amine chain was responsible for the major changes in the CD spectra and, as such, was considered fundamental for structuring.

The remaining polymers, M-G-*l*-Trp_10_, M-G-*l*-Trp_20_ and M-G-*l*-Trp_40_ proved to be less pH-responsive, probably due to the increase in tryptophan content, whose dipole moment and B_b_ transition balanced those of glycine repeating units, resulting in lower intensities. However, the same trend of the wavelength at which the molar ellipticity maxima occurred was observed with the pH. It may be observed that M-G-*l*-Trp_20_ and M-G-*l*-Trp_40_ spectra, at pH 8 and 11, corresponding, respectively, to 67% and 100% *tert*-amine deprotonation, were quite similar to those of M-*l*-Trp. They both presented a positive peak with equal or higher intensity than the negative one and seemed rather unaffected by pH. Upon increasing *l*-tryptophan content from M-G-*l*-Trp_5_ to M-*l*-Trp, negative peaks, at the same pH, resulted ≈10 nm shifted to higher wavelength, accompanied by reduced intensities. In contrast, the UV–Vis absorption spectra remained unmodified (Figure S6). This suggested the establishing of intramolecular interactions between chromophores. The lower intensity recorded for these negative peaks suggested a change in the distribution of the electronic density, associated with the balancing of the dipole moments of the randomly distributed tryptophan units. The positive peaks showed only intensity differences. In fact, as the content of tryptophan became higher, the major contribution was associated with the consequently predominant indole B_b_ transition.

The CD spectral pattern of M-*l*-Trp, with the highest tryptophan content, was similar to that of *l*-tryptophan, whose aromatic side chain generated only one strong positive transition at pH 7.0 [31].

### 3.6. Photoluminescence Analysis

**Figure 8:**
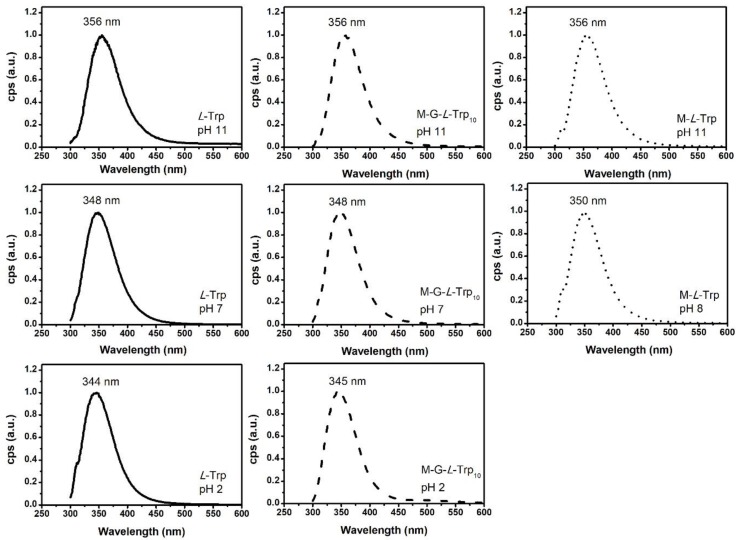
pH-dependence of the emission spectra of M-*l*-Trp and M-G-*l*-Trp_10_ compared with those of *l*-tryptophan at *λ*_ex_ = 279 nm. For the sake of clarity, some curves have been omitted in Figure S7.

The fluorescent properties of *l*-tryptophan are widely used to probe both conformational dynamics and microenvironment of proteins and peptides [32]. These properties originate from the two low-energy indole excited states, namely ^1^L_a_ and ^1^L_b_ [33,34,35,36,37]. The dipole moment of ^1^L_b_ is small and very close to that of the ground state (1.86 D), whereas the dipole moment of ^1^L_a_ is large (5.86 D), imparting *l*-tryptophan fluorescence high sensitivity to changes in the micro-environment [38]. In water, *l*-tryptophan exists in three ionization states. All of them are fluorescent [39,40], and their emission spectra are pH- [40] and solvent [41] sensitive. In the present work, photoluminescence preliminary studies were performed on the pH-dependence of *l*-tryptophan emission spectra and quantum yields. As expected from literature, *l*-tryptophan emission spectra shifted to higher wavelength with increasing pH (Figure 8 and Table S2), due to the different local electrostatic environment of the indole moiety [42]. 

Quantum yields (QY) measurements (Table 4) indicated that the highest value was reached for the anionic form (22.1%) at pH 11, followed by the zwitterionic form (11.7%) at pH 7, and by the cationic form (4.2%) at pH 2. This trend was ascribed to the different intramolecular quenching processes involving the indole excited states, that is, the positively-charged ammonium group and the carboxyl group. Time-resolved fluorescence measurements showed biexponential decays; hence, two lifetimes were determined (Table S3 and Figure S8), which is in agreement with literature data [43]. Lifetimes followed the same trend described for quantum yields and are normally interpreted by the rotamer model [43,44,45]. This model implies that, as the interconversion between rotamers is slower (ms) than the fluorescence time scale (ns), the fluorescence decay is multiexponential with relative amplitudes proportional to the rotamer populations. In particular, the different lifetimes of the rotamers arise from the different distances of the quenching functional groups from the indole moiety. At pH 7, the calculated lifetimes were 0.2 and 2.79 ns. The former was ascribed to the rotamer whose ammonium group was closer to the indole ring, whereas the latter was ascribed to rotamers whose carboxylate groups, a less efficient quencher, were closer to the indole ring [44]. Similar considerations explained the decrease, by decreasing pH, of *l*-tryptophan lifetimes (Table S3). In air, also O_2_-collisional quenching processes may occur [46,47]. To study the efficiency of O_2_ as a *l*-tryptophan quencher, QYs of degassed and non-degassed solutions at pH 11 were recorded (Table 4). As expected from the literature [46,47], the higher QY corresponded to degassed solutions.

In the present work, the fluorescence properties of M-*l*-Trp and M-G-*l*-Trp copolymers were studied at 2 × 10^−4^ M concentration, referred to the repeat units, in non-degassed ultrapure water and at different pH’s, corresponding to different charge distributions. The pH values considered were different for different tryptophan contents in the polymers, since M-*l*-Trp and M-G-*l*-Trp_40_ were soluble only at pH > 8. Excitation spectra were carried out at the polymer emission maxima (Table S2). 

The UV–Vis and excitation spectra of all tryptophan-based PAACs were superimposable to that of *l*-tryptophan, irrespective of tryptophan content (Figure S9) and pH (see Figure S10 for M-G-*l*-Trp_5_ reported as an example). 

The emission spectra (Figure 8 and Figure S7) recorded at 279 nm were unstructured and, at the same pH, almost superimposable to that of *l*-tryptophan. This provided solid evidence that M-*l*-Trp and M-G-*l*-Trp photoluminescence properties were governed solely by tryptophan. As in *l*-tryptophan, increasing pH induced in PAACs a slight increase of the emission wavelength maximum (*λ*_em_) (Table S2), due to the modification of microenvironment surrounding the indole ring induced by the different degree of ionization.

Absolute photoluminescence quantum yield measurements (Table 4) showed that, as a rule, the QY values decreased with decreasing pH. The observed decrease was ascribed to intramolecular quenching processes involving the excited state of indole. As hypothesized for peptides, three possible indole quenchers were identified for tryptophan-based polymers: main chain amide groups, protonated amines and carboxyl group [48,49,50,51]. At higher pH’s, higher QYs were observed, since the electron transfer from the indole to the carboxylate quenching groups was less efficient than, at lower pH’s, the transfer to both COOH and ammonium groups [44,48]. At all pH values, by increasing *l*-tryptophan content, a significant decrease in QY was recorded, reaching the lowest value in the case of M-*l*-Trp. Furthermore, these values were significantly lower than those of *l*-tryptophan also in the case of M-G-*l*-Trp_5_, characterized by the lowest tryptophan unit content. Probably, at higher tryptophan contents, the proximity among *l*-tryptophan moieties maximized the quenching due to tryptophan-to-tryptophan homotransfer (resonance energy transfer) [52,53,54].

The absolute QYs of degassed and non-degassed pH 11 M-*l*-Trp and M-G-*l*-Trp solutions were compared to assess the efficiency of O_2_ as a quencher (Table 4). Unexpectedly, no significant differences were detected, in contrast to what observed with *l*-tryptophan. This was possibly due to the low accessibility of O_2_ to *l*-tryptophan bearing units for conformational reasons [55]. 

**Figure 9:**
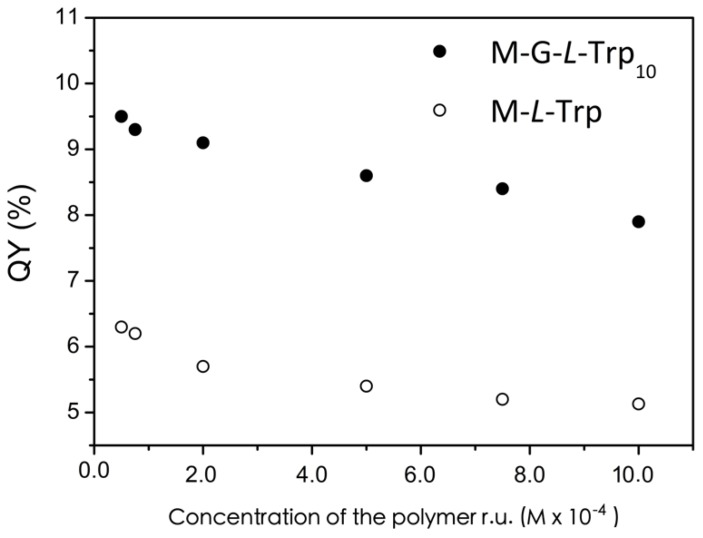
Quantum yields of M-*l*-Trp and M-G-*l*-Trp_10_ recorded at pH 11 and increasing concentration of the polymer repeat units (r.u.).

To evaluate intermolecular tryptophan quenching by approaching chains, the concentration effect was studied. Non-degassed solutions of M-*l*-Trp and M-G-*l*-Trp_10_ were studied at pH 11 in the 1 × 10^−3^–1 × 10^−5^ M concentration range of the repeat units (Figure 9). Although the two curves exhibited different absolute QY values, their trends were similar. In both cases, the QY values decreased with increasing concentration, suggesting quenching mechanism related to intermolecular interactions.

Time-resolved fluorescence measurements were performed on both M-*l*-Trp and M-G-*l*-Trp copolymers as a function of pH, at 2 × 10^−4^ M concentration referred to the repeat units and *λ*_ex_ = 301 nm. As previously observed for *l*-tryptophan, two lifetimes—a short (0.8–1.2 ns) and a long one (3.62–6.13 ns)—were determined (Figures S11–S15). Both lifetimes decreased with decreasing pH and increasing tryptophan content of the polymer samples (Table S3).

## 4. Conclusions

New tryptophan-based PAACs were synthesized by Michael-type polyaddition of MBA with *l*-tryptophan and *l*-tryptophan/glycine mixtures. The acid-base properties, as well as the self-structuring in aqueous solution of the resultant polymers, were studied. They showed composition- and pH-dependent solubility, as ascertained from UV–Vis absorption and scattering of polarized IR beam tests. DLS measurements in 0.1 M NaCl gave hydrodynamic radii stable at 25 °C for at least 1 month and unaffected by pH in the range 1–11 in case of glycine rich samples, and in the pH range 7–11 for M-G-*l*-Trp_40_ and M-*l*-Trp homopolymers. However, they were, to some extent, sensitive to concentration in the range 1–30 mg·mL^−1^. In water, all MBA-tryptophan PAACs showed CD spectra revealing pH-dependent self-structuring in the wavelength interval 200–280 nm. Photoluminescence measurements showed that all polymers exhibited pH-dependent quantum yield and lifetime of the excited states, as well as wavelength of the emission maximum. A significant intermolecular quenching by approaching chains was observed for tryptophan-rich samples. Chiral tryptophan-containing PAACs share the ability to self-structure in water with PAACs bearing arginine [22] and hydrophobic [23] side chains. 

It can be reasonably concluded that tryptophan-containing PAACs, combining chirality, multifunctionality, pH-dependent water solubility, self-structuring in water, chiro-optical and fluorescence properties, represent singular examples of synthetic bioinspired chiral polymers and can open an interesting field of investigation on account of their selective interactions with chiral structures, including biological structures.

## Figures and Tables

**Table 1:** Comparison of copolymers tryptophan content from reaction recipes and ^1^H NMR.

Sample	Tryptophan Content in the Feed ^a^	Tryptophan Content from ^1^H NMR ^a^
M-G-*l*-Trp_5_	5	4.53
M-G-*l*-Trp_10_	10	9.70
M-G-*l*-Trp_20_	20	17.4
M-G-*l*-Trp_40_	40	40.5

^a^ Expressed as mole %.

**Table 2:** Homo- and copolymers *M*_n_ evaluation by NMR and SEC: comparison between calculated and experimental *M*n values.

Sample	*M*_n_ from ^1^H NMR ^a^	*M*_n_ from ^1^H NMR ^b^	*M*_n_ from SEC
M-G-*l*-Trp_5_	12,000	6000	9000
M-G-*l*-Trp_10_	8800	4400	8800
M-G-*l*-Trp_20_	7600	3800	8600
M-G-*l*-Trp_40_	5700	2850	8300
M-*l*-Trp	5400	2700	-- ^c^

^a^ Calculated assuming terminal acrylamide units at both chain ends, ^b^ Calculated assuming terminal acrylamide units at one chain end. ^c^ Analyses were not carried out due to solubility limits.

**Table 3:** *pK*_a_ and *β* values of M-G-*l*-Trp copolymers in the *α* range 0.2–0.8 (*β*_1_) and 0.1−0.9 (*β*_2_).

Sample	*pK*_a1_	*pK*_a2_	*β*_1_	*β*_2_	IP
M-G-*l*-Trp_5_	2.05 ± 0.15	7.78 ± 0.12	0.61 ± 0.08	1.39 ± 0.03	4.9
M-G-*l*-Trp_10_	2.06 ± 0.18	7.75 ± 0.18	0.61 ± 0.06	1.36 ± 0.06	4.9
M-G-*l*-Trp_20_	2.04 ± 0.19	7.74 ± 0.13	0.57 ± 0.05	1.60 ± 0.11	4.9
M-G-*l*-Trp_40_	-	7.77 ± 0.02^c^	-	1.51 ± 0.07	-

^a^ Carboxyl group. ^b^ Average values obtained from four forward titration experiments. ^c^ Amine group. ^d^ Isoelectric point. ^e^ Calculated from the simulated titration curves by the De Levie approach.

**Table 4:** Quantum yields of *l*-tryptophan, M-G-*l*-Trp and M-*l*-Trp copolymers at different pH’s. All values should be considered with a ± 5% S.D.

pH	*l*-tryptophan	M-G-*l*-Trp_5_	M-G-*l*-Trp_10_	M-G-*l*-Trp_20_	M-G-*l*-Trp_40_	M-*l*-Trp
**11**	23.0 28.4 ^a^	14.3 15.2 ^a^	9.5 11.0 ^a^	7.6 7.8 ^a^	7.2 7.5 ^a^	6.3 6.1 ^a^
**7**	11.7	8.5	6.4	5.7	4.9 ^b^	4.0 ^b^
**2**	4.9	5.6	4.7	4.9	2.0 ^b^	-

^a^ Analyses carried out after three freeze-pump-thaw cycles. ^b^ Analyses carried out at pH 1.5–8, instead of 2 and 7, due to solubility limits.

## Supplementary Materials

The following are available online at https://www.mdpi.com/2073-4360/11/3/543/s1, Figure S1: Titration and speciation curves referred to the 1^st^ experiment of Table S1 for M-G-*l*-Trp_5_, M-G-*l*-Trp_10_, M-G-*l*-Trp_20_ and M-G-*l*-Trp_40_: experimental, simulated and *β* corrected titrations (a); distribution of charged species (b). The speciation curve of M-G-*l*-Trp_40_ is calculated assuming *pKa_1_* = 2.00; Figure S2: Determination of *β* parameters for side –COOH and chain *tert*-amine of M-G-*l*-Trp_5_, M-G-*l*-Trp_10,_ M-G-*l*-Trp_20_ and M-G-*l*-Trp_40_ referred to the 1^st^ experiment of Table S1: calculation of *β* values from Eq. 1b (a); trend of the *β*-corrected *pK_a_* values vs *α* according to Eq. S1a (b); Figure S3: ^1^H-NMR spectra recorded in D_2_O; Figure S4: SEC analyses (refractive index signal) of *l*-tryptophan-based copolymers, in 0.1 M Tris buffer (pH 8.00 ± 0.05) solution with 0.2 M sodium chloride; Figure S5: FTIR-ATR spectra of the investigated *l*-tryptophan-based homo- and copolymers; Figure S6: Circular dichroism and UV-vis absorption spectra of *l*-tryptophan-based homo- and copolymers, at pH 11; Figure S7: pH dependence of M-G-*l*-Trp_5_, M-G-*l*-Trp_20_ and M-G-*l*-Trp_40_ emission spectra recorded at *λex* = 279 nm and 25 °C; Figure S8: Emission decay of *l*-Trp vs pH at *λex* = 301 nm: data (black line), instrument response function (IRF) (blue line) and convolution fit (red line). Weighted residuals are shown under the decay curves; Figure S9: *l*-Tryptophan-based homo- and copolymers spectra recorded at pH 11: a) excitation (*λem* = 356 nm) and b) UV-vis absorption. *l*-Tryptophan is reported for comparison purposes as well; Figure S10: pH-dependence of M-G-*l*-Trp_5_ spectra: a) excitation (*λem* = 356 nm) and b) UV-vis absorption; Figure S11: Emission decay of M-G-*l*-Trp_5_ vs pH at *λex* = 301 nm: data (black line), instrument response function (IRF) (blue line) and convolution fit (red line). Weighted residuals are shown under the decay curves; Figure S12: Emission decay of M-G-*l*-Trp_10_ vs pH at *λex* = 301 nm: data (black line), instrument response function (IRF) (blue line) and convolution fit (red line). Weighted residuals are shown under the decay curves; Figure S13: Emission decay of M-G-*l*-Trp_20_ vs pH at *λex* = 301 nm: data (black line), instrument response function (IRF) (blue line) and convolution fit (red line). Weighted residuals are shown under the decay curves; Figure S14: Emission decay of M-G-*l*-Trp_40_ vs pH at *λex* = 301 nm: data (black line), instrument response function (IRF) (blue line) and convolution fit (red line). Weighted residuals are shown under the decay curves; Figure S15: Emission decay of M-*l*-Trp vs pH at *λex* = 301 nm: data (black line), instrument response function (IRF) (blue line) and convolution fit (red line). Weighted residuals are shown under the decay curves. Table S1: *l*-Tryptophan-based copolymers’ *pK_a_* values, calculated from forward titration data taking into accounts solubility limits. M-G-*l*-Trp_40_ *pK_a_* values are obtained from back-titration; Table S2: Emission maximum of *l*-tryptophan, homo- and copolymers recorded by steady-state fluorescence measurements of non-degassed solutions in distilled water vs pH at *λex* = 279 nm; Table S3: *l*-tryptophan, homo- and copolymers time-resolved fluorescence measurements of non-degassed solutions in distilled water versus pH. In parentheses % of *l*-tryptophan populations that decay at the calculated *τ* time. *λ_ex_* = 301 nm.
