# Supplementary material for: d-, l- and d,l-Tryptophan-Based Polyamidoamino Acids: pH-Dependent Structuring and Fluorescent Properties
Source: Polymers (Basel). 2019 Mar 22;11(3):543. doi: 10.3390/polym11030543 (PMC6473350; doi:10.3390/polym11030543)
Supplement: Supplementary file 1 [file polymers-11-00543-s001.pdf]

Supplementary Materials

D-, L- and D,L-Tryptophan-Based Polyamidoamino Acids: pH-Dependent Structuring and Fluorescent Properties

Federica Lazzari [1,*], Amedea Manfredi [1], Jenny Alongi [1], Daniele Marinotto [2], Paolo Ferruti [1,*] and Elisabetta Ranucci [1,*]

[1] Dipartimento di Chimica, Università degli Studi di Milano, via C. Golgi 19, 20133 Milano, Italy; amedea.manfredi@unimi.it (A.M.); jenny.alongi@unimi.it (J.A.)
[2] Istituto di Scienze e Tecnologie Molecolari (ISTM-CNR), via C. Golgi 19, 20133 Milano, Italy; daniele.marinotto@istm.cnr.it

Correspondence: federica.lazzari@unimi.it (F.L.); paolo.ferruti@unimi.it (P.F.); elisabetta.ranucci@unimi.it (E.R.); Tel.: +39–02-5031-4128 (P.F.); +39–02-5031-4132 (E.R.)

Determination of pK_a values, β parameters and speciation curves

^{1}H-NMR

Size Exclusion Chromatography (SEC)

FTIR-ATR spectra

UV-vis and circular dichroism spectroscopy

Photoluminescence and life time analysis

Figure S1-S15

Tables S1-S3

Reference

Determination of pKa values, β parameters and speciation curves

pKa determination. The pK_{a1} (side -COOH) and pK_{a2} (chain *tert*-amine) values of the ionizable functions present in the studied PAACs were determined as the pH values at the half-equivalent points, located in the buffer zone related to the specific function. The half-equivalent points were obtained as the half-titrant volume amounts added between consecutive inflections in the pH versus titrant volume curves. The inflection points were in turn determined by numerically calculating the second derivative of the pH versus volume curves (Figure S1).

β parameter determination. The β parameters of the generalized Henderson-Hasselbalch equation (Eq. S1a) were determined for both pK_{a1} (side -COOH) and pK_{a2} (chain *tert*-amine) to ascertain the presence of interactions between ionizable groups on adjacent monomeric units. The β parameters were determined by firstly selecting the specific buffer region intervals marked by each pK_a. The dissociation degree, α, was then calculated in each zone as the ratio between the reacted moles and the total amount of moles necessary to reach complete neutralization. β Values were finally obtained from Eq. S1b as the slope of the pH versus –log((1-α)/α) curve (Figures S2a). Points near inflections deviated from ideality and were not considered. Figures S2b shows the β-corrected pK_a values in the chosen α intervals.

$$pK_a = pH + \beta \times \log\frac{1-\alpha}{\alpha} \quad \text{(Eq. S1a)} \qquad \leftrightarrow \qquad pH = pK_a - \beta \times \log\frac{1-\alpha}{\alpha} \quad \text{(Eq. S1b)}$$

Determination of simulated titration curves. Simulated titration curves were determined following the De Levie approach [1] in order to iteratively refine pKa and β values to achieve the best fitting to the experimental data.

- <u>Initial conditions:</u>

V_0 = initial solution volume
c_0 = initial PAACs concentration expressed as molarity of the repeat unit
c_S = initial concentration of ionic strength stabilizer
c_t = titrant concentration (strong base in forward titration or acid in backward titration)
V_t = volume of the titrant added (strong base in forward titration or acid in backward titration)
c_A or c_B = acid concentration (or base in backward titration) used to correct pH
N = moles of strong acid possibly present as residual from the synthetic process or PAACs pretreatments

- *Mass balance:*

$$C_{PAACS} = C_{L^+} + C_{L^0} + C_{L^-} = \frac{C_0V_0}{V_0+V_t} \qquad \text{(Eq. S2)}$$

- *Equilibrium constants* (Eq. S3a-c):

$$K_{a1} = \frac{C_{L^0}C_{H^+}}{C_{L^+}} \text{ (a);} \quad K_{a2} = \frac{C_{L^-}C_{H^+}y^2}{C_{L^0}} \text{ (b);} \quad K_w = C_{H^+}C_{OH^-}y^2 \text{ (c);}$$

- *Concentration fractions* (Eq. S4a-c):

$$\alpha_2 = \frac{C_{L^+}}{C} = \frac{C_{H^+}^2}{D} \text{ (a);} \quad \alpha_1 = \frac{C_{L^0}}{C} = \frac{C_{H^+}y^2K_{a1}}{D} \text{ (b);} \quad \alpha_0 = \frac{C_{L^-}}{C} = \frac{K_{a1}K_{a2}}{D} \text{ (c);}$$

with:

$$\mathrm{D} = \mathrm{C}_{\mathrm{H}^+}^2 + \mathrm{C}_{\mathrm{H}^+}\mathrm{K}_{\mathrm{a}1} + \mathrm{K}_{\mathrm{a}1}\mathrm{K}_{\mathrm{a}2} \qquad \text{(Eq. S5)}$$

The activity coefficients (Davies equation):

$$y = 10^{-0.5\left[\frac{\sqrt{I}}{1+\sqrt{I}}-0.3I\right]} \qquad \text{(Eq. S6)}$$

Ionic strength:

$$I = \frac{1}{2}(C_{H^+} + C_{OH^-} + C_{Na^+} + C_{Cl^-} + C_{L^+} + C_{L^-}) \qquad \text{(Eq. S7)}$$

- *Charge balance*:

$$H^+ + Na^+ + L^+ = L^- + OH^- + Cl^- \text{ (Eq. S8)}$$

where (Eq. S9a-e):

$$C_{Na^+} = \frac{C_TV_T+C_SV_0}{V_0+V_T} \text{ (a);} C_{Cl^-} = \frac{C_SV_0+C_AV_A+N}{V_0+V_T} \text{ (b);} \quad C_{L^+} = \frac{\alpha_2C_0V_0}{V_0+V_T} \text{ (c);}$$

$$C_{L^-} = \frac{\alpha_0C_0V_0}{V_0+V_T} \text{ (d);} \quad C_{OH^-} = \frac{K_w}{C_{H^+}y^2} \text{ (e);}$$

Combining all former conditions, the following solving equation, representing the whole forward titration curve, was obtained in terms of V_T as a function of pH:

$$V_T = \frac{V_0[C_0(\alpha_0-\alpha_2)+C_A-\Delta]+N}{\Delta+C_T} \qquad \text{(Eq. S10)}$$

where:

$$\Delta = H^+ - OH^- = H^+ - {}^{K_w}/_{H^+y^2} \qquad \text{(Eq. S11)}$$

The whole backward titration is expressed in terms of V_T as a function of pH:

$$V_T = \frac{V_0[C_0(\alpha_0 - \alpha_2) + C_A - \Delta] + N - (\Delta + C_B)V_B}{\Delta - C_T} \qquad \text{(Eq. S12)}$$

Simulated titration curves, reported in Figure S1, were obtained from Eq. S10-S12 in the buffer regions relative to both side -COOH and *tert*-amine groups. Comparison between pK_a and β-corrected pK_a values is reported. Calculation were carried out considering C_{Na^+} and C_{Cl^-} constant throughout the whole titration experiment and equal to 0.1 M. Concentration fractions α and pK_a values were refined iteratively to achieve the best fitting to the experimental points.

Determination of speciation diagrams. Speciation diagrams were obtained by plotting the concentration fractions of the differently ionic species as a function of pH (Eq. S12a-c):

$$\alpha_2 = \frac{C_{L^-}}{C} = \frac{C_{H^+}^2}{D} \qquad \text{Eq. S12a}$$

$$\alpha_1 = \frac{C_{L^0}}{C} = \frac{C_{H^+} y^2 K_{a1}}{D} \qquad \text{Eq. S12b}$$

$$\alpha_0 = \frac{C_{L^-}}{C} = \frac{K_{a1} K_{a2}}{D} \qquad \text{Eq. S12c}$$

With D and y as previously described, and where the K_{a1} and K_{a2} values were corrected for β_1 and β_2.

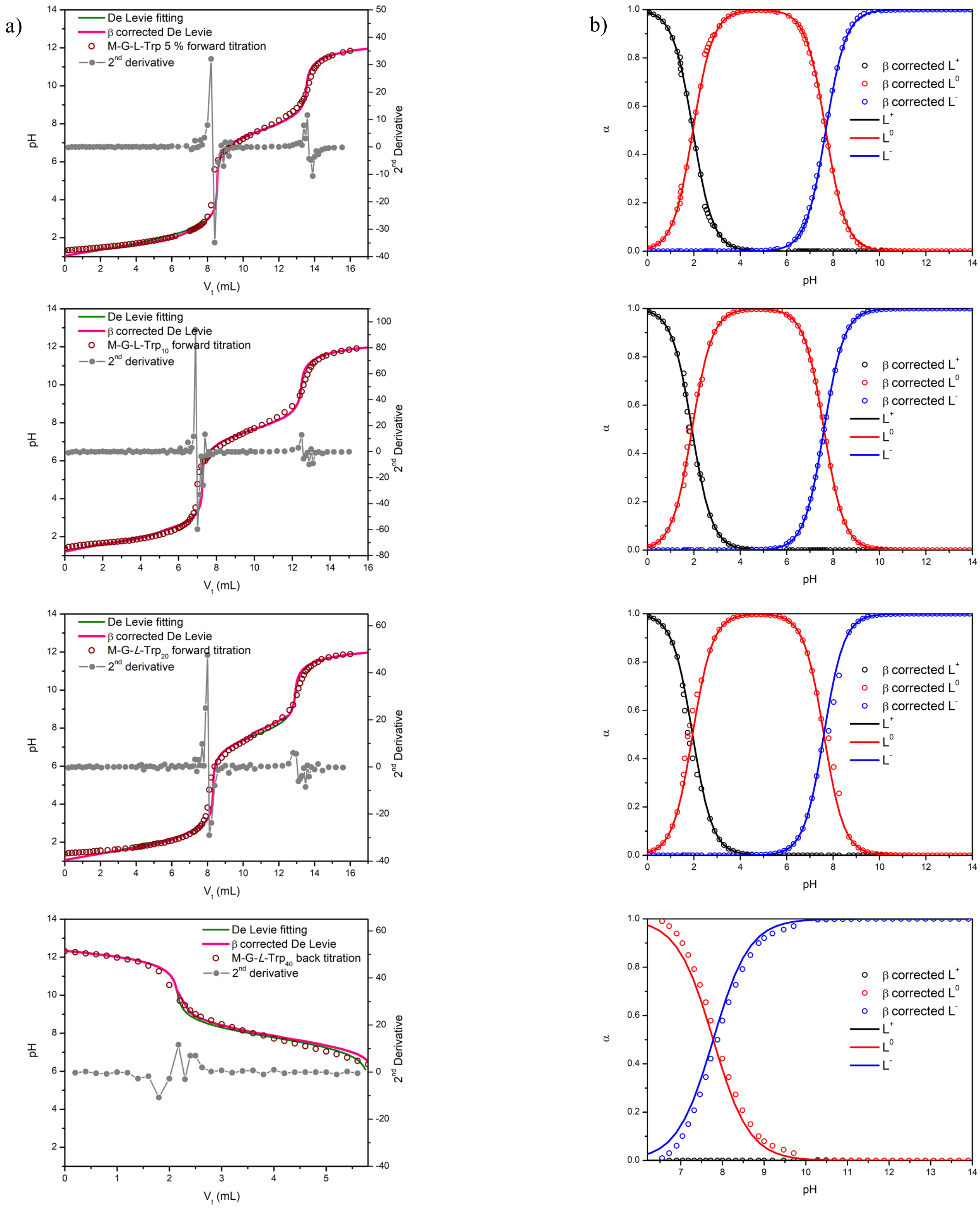

Figure S1. Titration and speciation curves referred to the 1st experiment of Table S1 for M-G-*L*-Trp$_5$, M-G-*L*-Trp$_{10}$, M-G-*L*-Trp$_{20}$ and M-G-*L*-Trp$_{40}$: experimental, simulated and β corrected titrations (a); distribution of charged species (b). The speciation curve of M-G-*L*-Trp$_{40}$ is calculated assuming $pKa_1 = 2.00$.

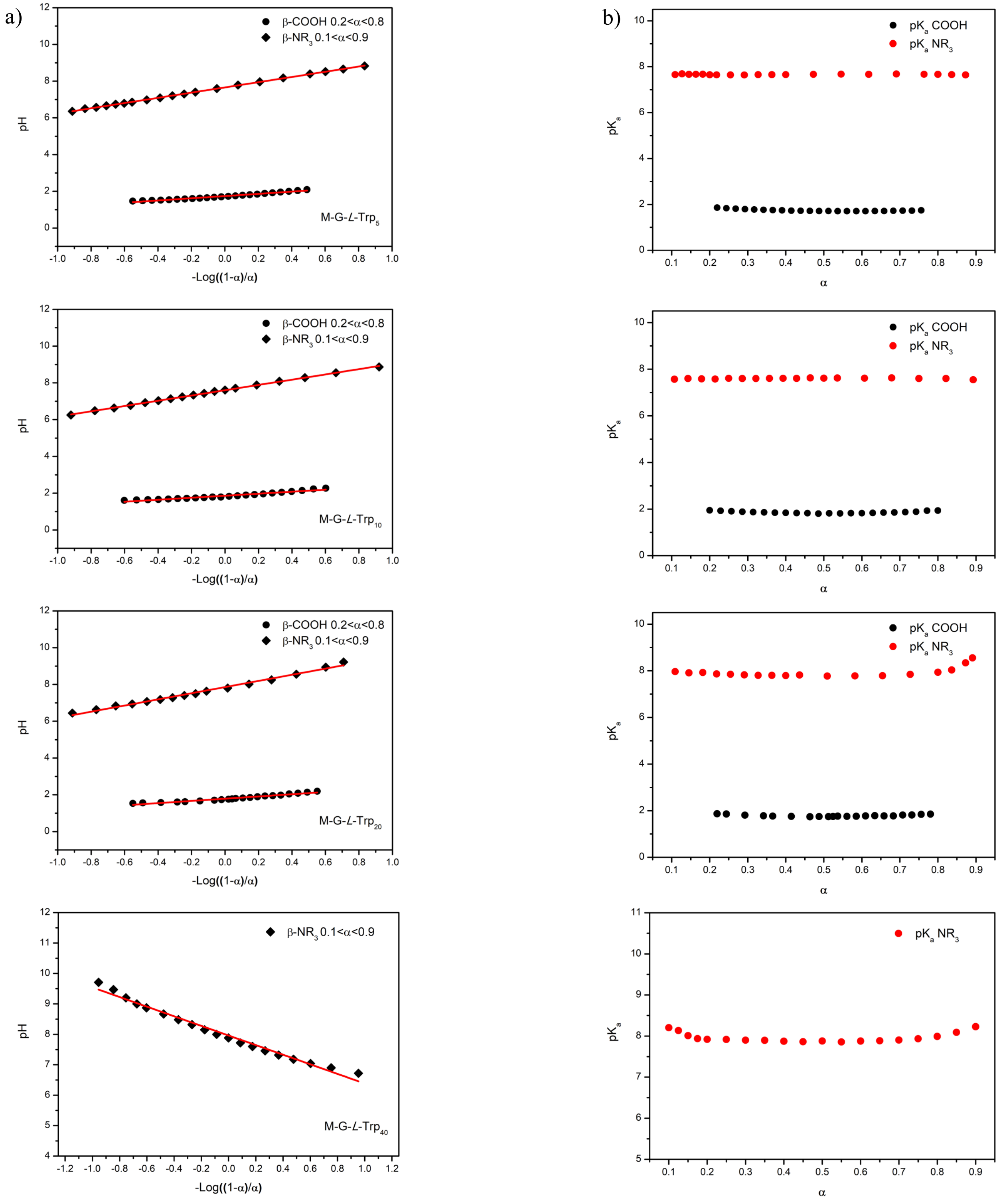

Figure S2. Determination of β parameters for side –COOH and chain *tert*-amine of M-G-*L*-Trp$_5$, M-G-*L*-Trp$_{10}$, M-G-*L*-Trp$_{20}$ and M-G-*L*-Trp$_{40}$ referred to the 1st experiment of Table S1: calculation of β values from Eq. 1b (a); trend of the β-corrected pK_a values vs α according to Eq. S1a (b).

PAAC	^{1}H-NMR spectrum
**M-*L*-Trp**<br><br>δ (ppm) 2.01-2.08 (A), 2.33-2.93 (B,C), 3.86 and 4.16-4.27 (D), 3.93-4.00 (E), 6.90-7.06 (F,G,H), 7.21-7.52 (I,L).	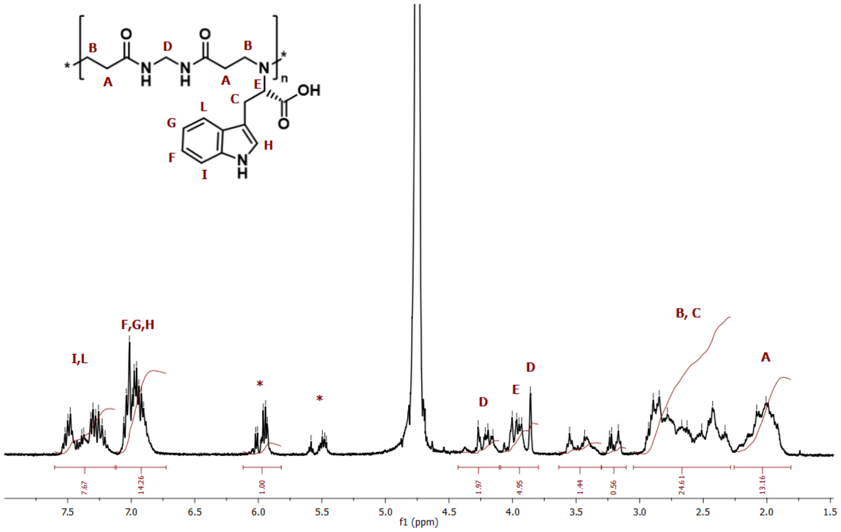
**M-G-*L*-Trp$_5$**<br><br>δ (ppm) 2.19-2.22 (A), 2.32 (A'), 2.61-2.62 (B), 2.73-2.76 (B'), 2.93 (C), 3.04 (D), 4.29 (E), 4.35 (F), 4.44-4.47 (F,F'), 7.04-7.13 (G,H,I), 7.36-7.40 (L), 7.60-7.62 (M).	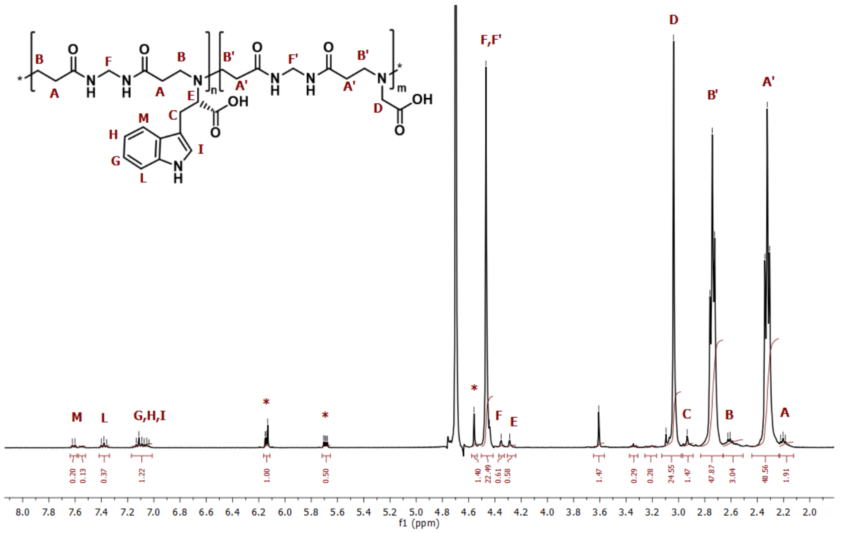
**M-G-*L*-Trp$_{10}$**<br><br>δ (ppm) 2.19-2.22 (A), 2.31-2.34 (A'), 2.61-2.62 (B), 2.73-2.76 (B'), 2.93 (C,D), 3.04 (E), 4.35 (F), 4.47 (F'), 7.05-7.13 (G,H,I), 7.36-7.38 (L), 7.54-7.62 (M).	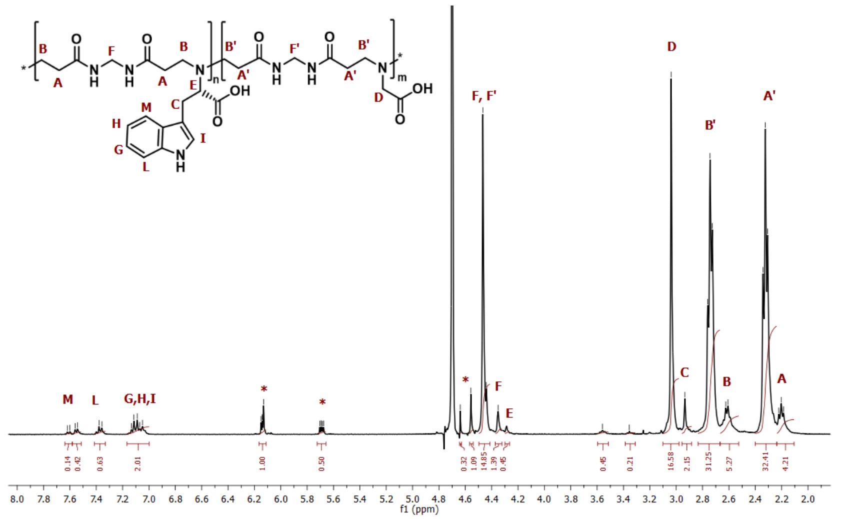

M-G-*L*-Trp$_{20}$

δ (ppm) 2.19-2.22 (A), 2.30-2.34 (A’), 2.59-2.62 (B), 2.72-2.74 (B’), 2.93 (C), 3.04 (D), 4.29 (E), 4.35 (F), 4.44-4.47 (F,F’), 7.05-7.13 (G,H,I), 7.36-7.38 (L), 7.54-7.60 (M).

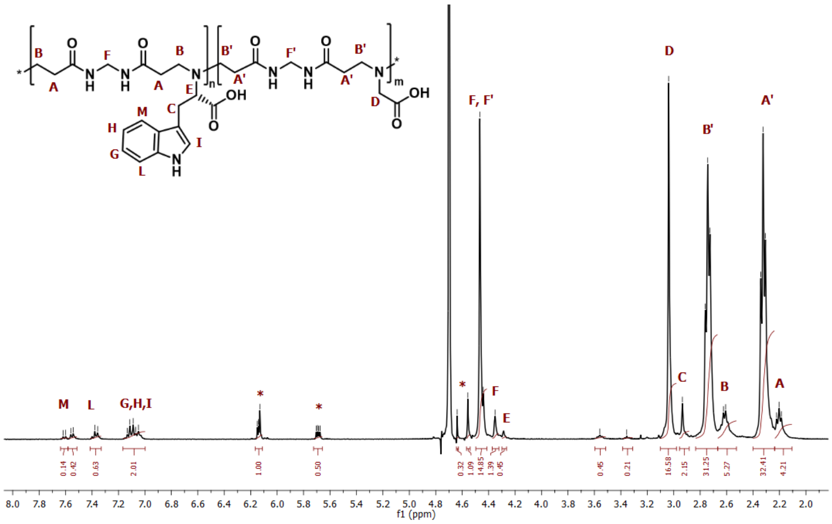

M-G-*L*-Trp$_{40}$

δ (ppm) 2.11 (A), 2.22 (A’), 2.51 (B), 2.65 (B’), 2.86 (C), 2.96 (D), 4.06-4.23 (E,F), 4.39-4.47 (F,F’), 6.98-7.02 (G,H,I), 7.30 (L), 7.45-7.55 (M).

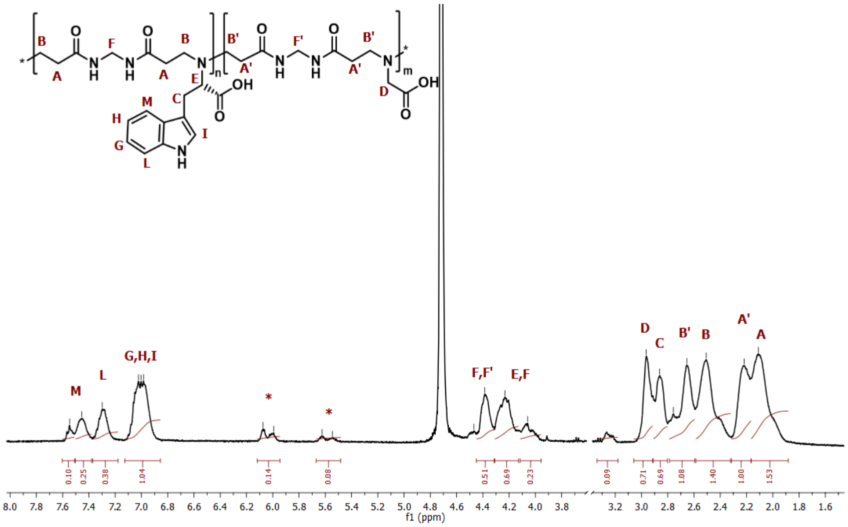

Figure S3. ^{1}H-NMR spectra recorded in D_2O.

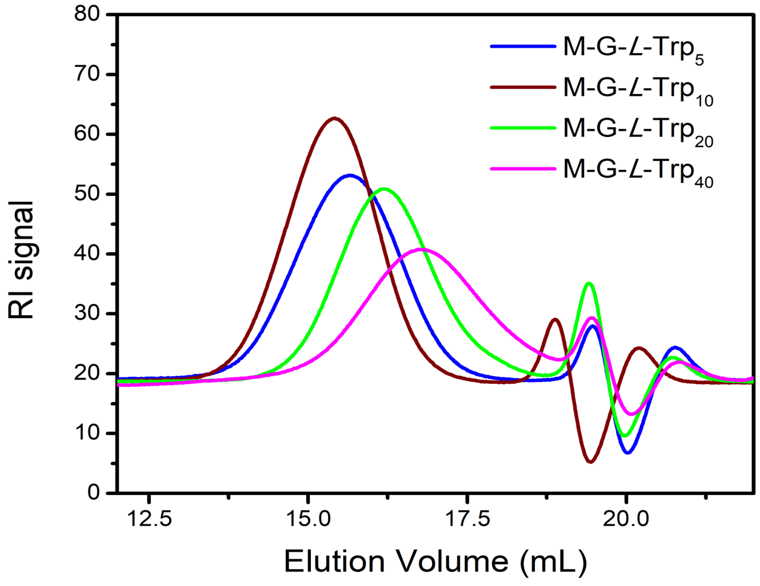

Figure S4. SEC analyses (refractive index signal) of *L*-tryptophan-based copolymers, in 0.1 M Tris buffer (pH 8.00 ± 0.05) solution with 0.2 M sodium chloride.

Fourier-transform infrared spectroscopy (FTIR) analysis. FTIR in attenuated total reflectance configuration (ATR) spectra were recorded performing 16 scans at 4 cm^{-1} resolution in the 4000-500 cm^{-1} range, using a Perkin Elmer Spectrum 100 spectrometer equipped with a diamond crystal (penetration depth = 1.66 μm). Before each analysis, all samples were dried to constant weight under vacuum.

PAAC	FTIR-ATR spectrum

M-*L*-Trp

Signal	Wavenumber (cm^{-1})	Assignment
**1**	3252-3257	N-H stretching
**2**	3057-3059	C-H stretching
**3**	1629-1635	C=O stretching
**4**	1533-1542	N-H bending
**5**	1338-1342	O-H bending
**6**	1225-1228	C-N stretching
**7**	1190-1193	C-O stretching
**8**	1041-1047	C-C stretching
**9**	744	C-H vibrations out of the plane of aromatic rings in Trp

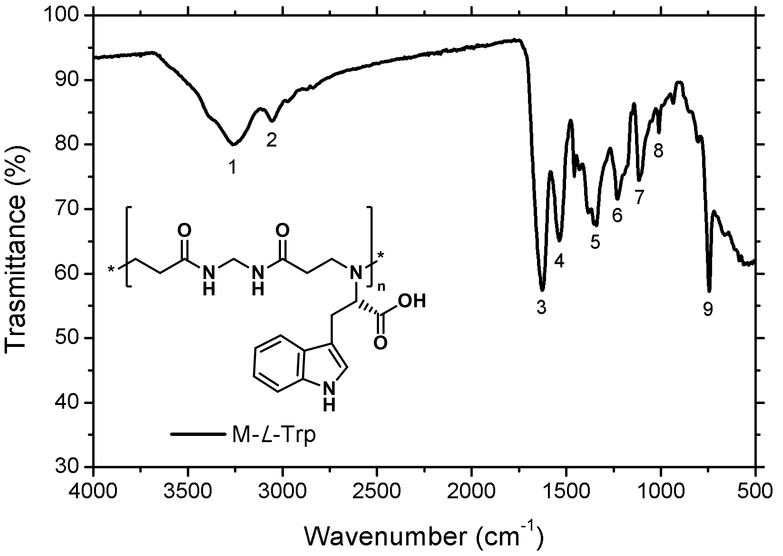

M-G-*L*-Trp_5

Signal	Wavenumber (cm^{-1})	Assignment
**1**	3247-3260	N-H stretching
**2**	3045-3057	C-H stretching
**3**	1617-1625	C=O stretching
**4**	1533-1537	N-H bending
**5**	1382-1387	O-H bending
**6**	1328-1343	C-H bending
**7**	1224-1229	C-N stretching
**8**	1185-1190	C-O stretching
**9**	1114-1118	C-C stretching
**10,11**	955-958 and 908-914	C-H vibrations out of the plane in Gly

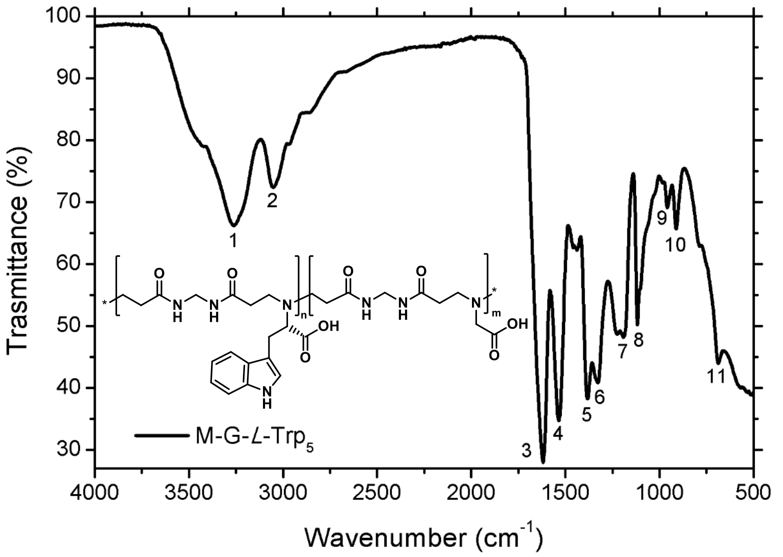

M-G-*L*-Trp$_{10}$

Signal	Wavenumber (cm^{-1})	Assignment
**1**	3247-3260	N-H stretching
**2**	3045-3057	C-H stretching
**3**	1617-1625	C=O stretching
**4**	1533-1537	N-H bending
**5**	1382-1387	O-H bending
**6**	1328-1343	C-H bending
**7**	1224-1229	C-N stretching
**8**	1185-1190	C-O stretching
**9**	1114-1118	C-C stretching
**10,11**	955-958 and 908-914	C-H vibrations out of the plane in Gly

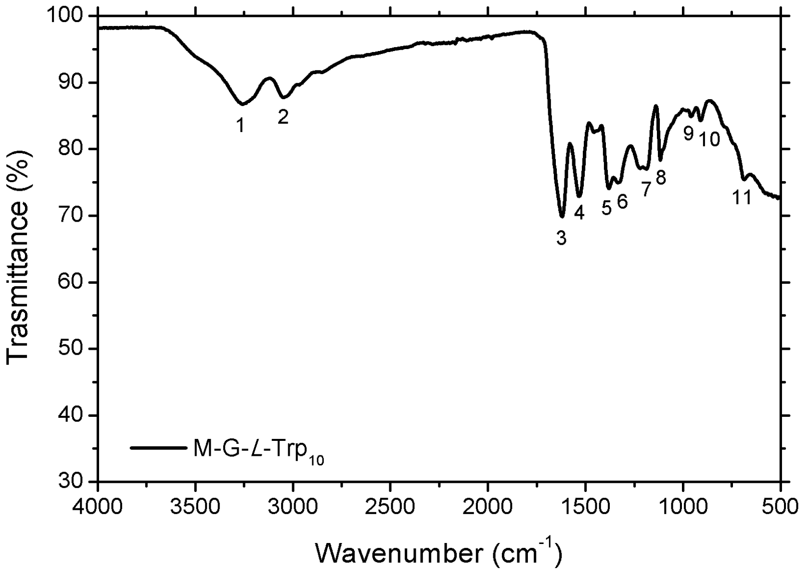

M-G-*L*-Trp$_{20}$

Signal	Wavenumber (cm^{-1})	Assignment
**1**	3247-3260	N-H stretching
**2**	3045-3057	C-H stretching
**3**	1617-1625	C=O stretching
**4**	1533-1537	N-H bending
**5**	1382-1387	O-H bending
**6**	1328-1343	C-H bending
**7**	1224-1229	C-N stretching
**8**	1185-1190	C-O stretching
**9**	1114-1118	C-C stretching
**10,11**	955-958 and 908-914	C-H vibrations out of the plane in Gly
**12**	742-744	C-H vibrations out of the plane of aromatic rings in Trp

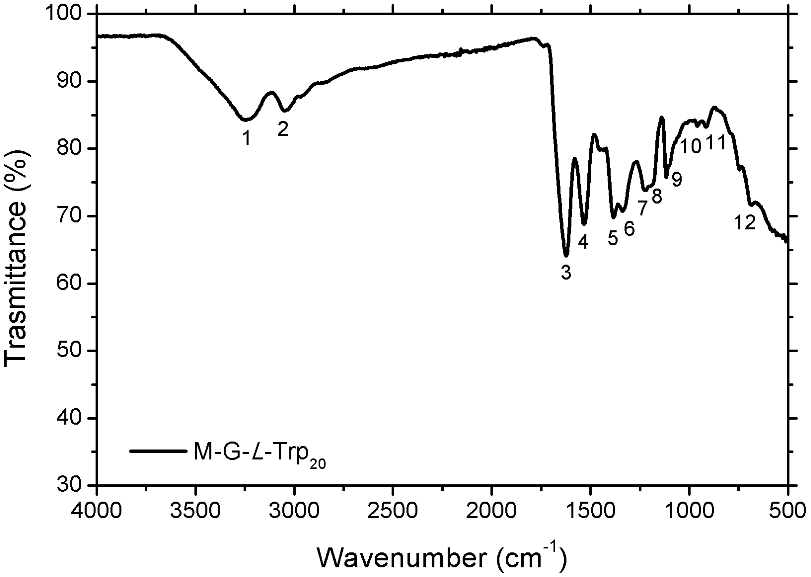

M-G-*L*-Trp40

Signal	Wavenumber (cm^{-1})	Assignment
**1**	3247-3260	N-H stretching
**2**	3045-3057	C-H stretching
**3**	1617-1625	C=O stretching
**4**	1533-1537	N-H bending
**5**	1382-1387	O-H bending
**6**	1328-1343	C-H bending
**7**	1224-1229	C-N stretching
**8**	1185-1190	C-O stretching
**9**	1114-1118	C-C stretching
**10,11**	955-958 and 908-914	C-H vibrations out of the plane in Gly

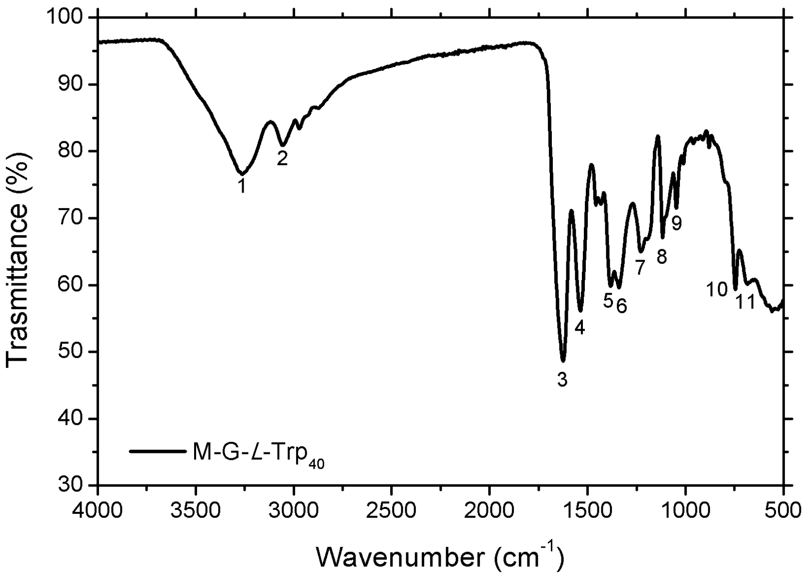

Figure S5. FTIR-ATR spectra of the investigated *L*-tryptophan-based homo- and copolymers.

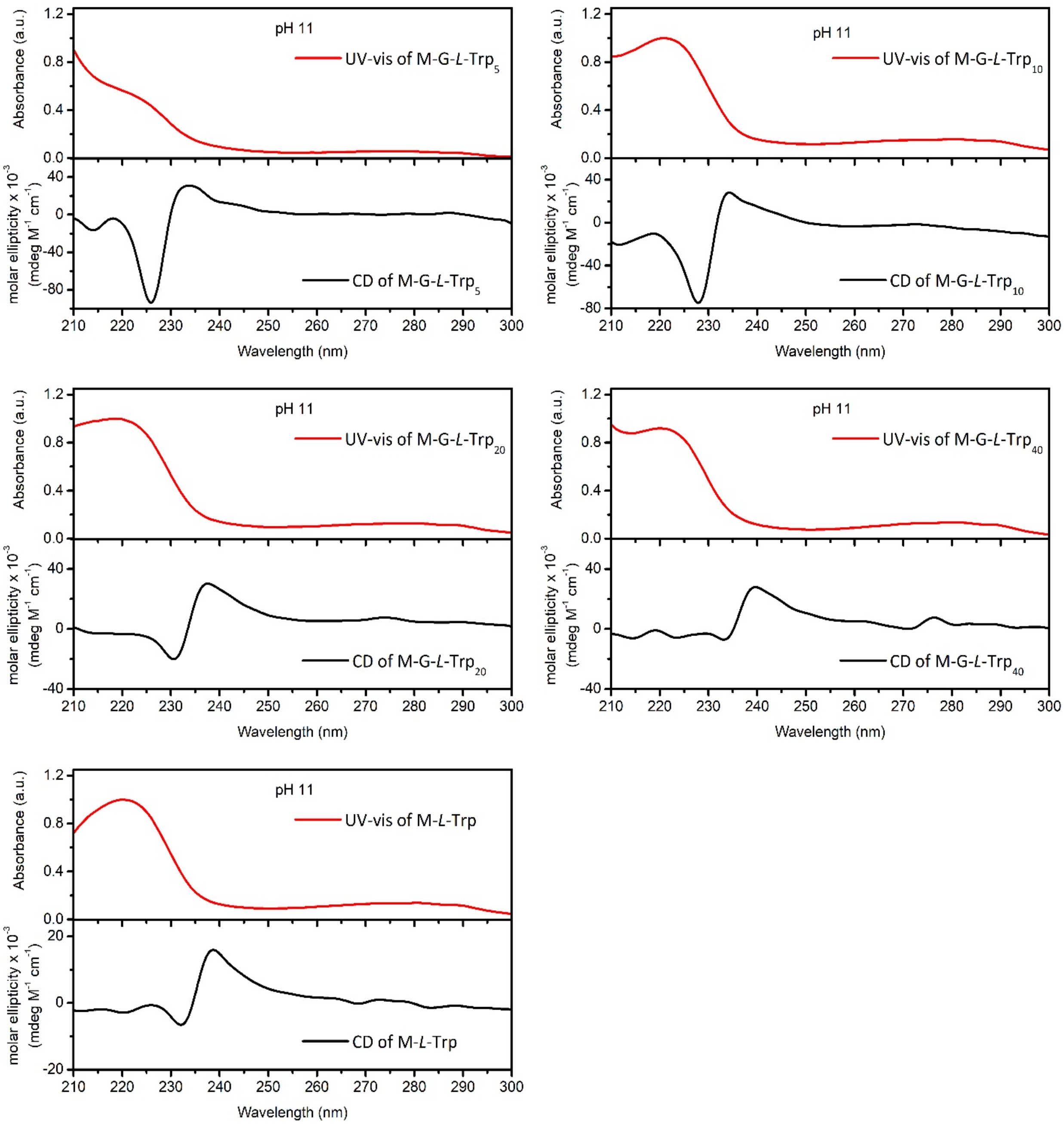

Figure S6. Circular dichroism and UV-vis absorption spectra of *L*-tryptophan-based homo- and copolymers, at pH 11.

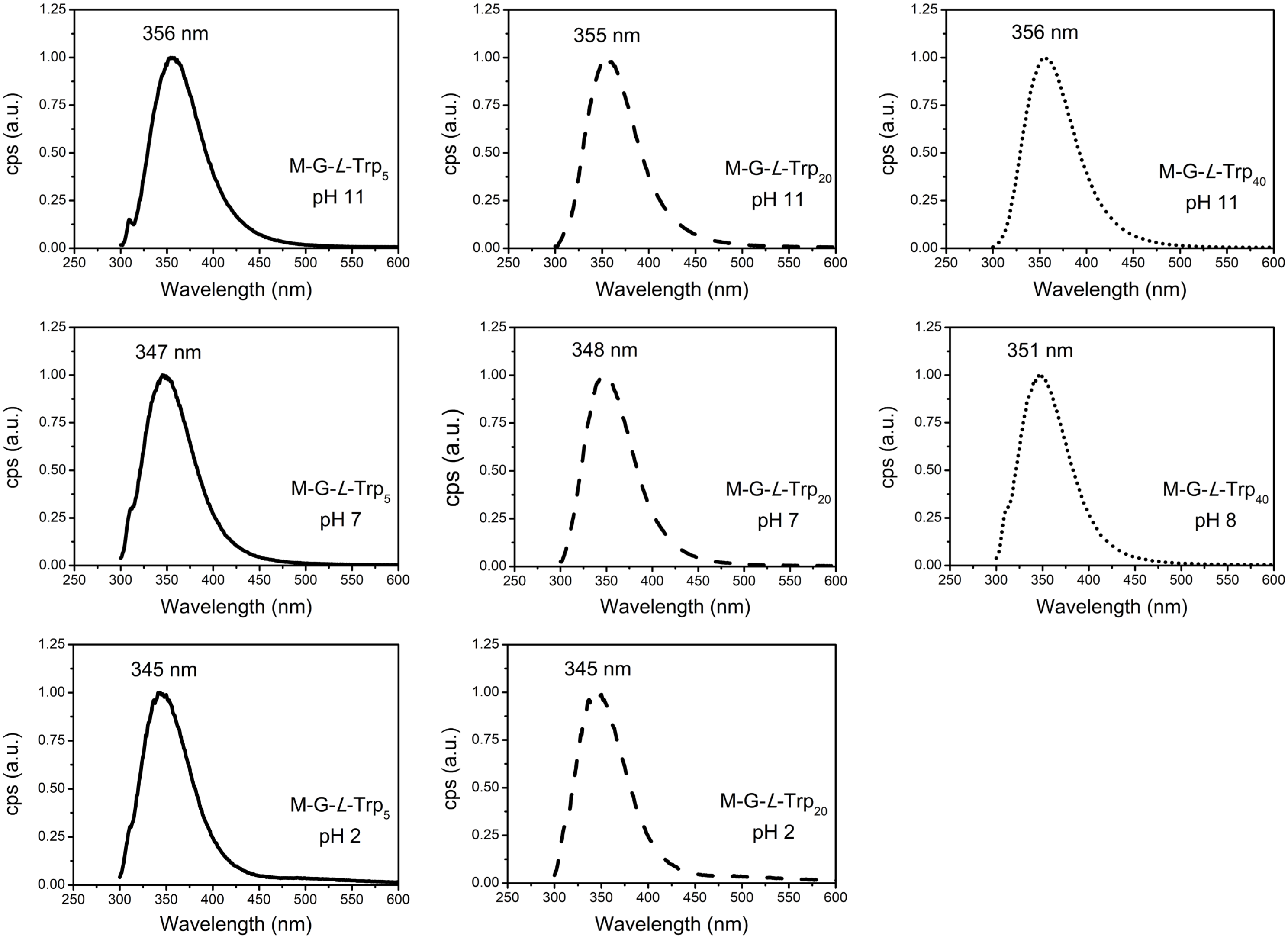

Figure S7. pH dependence of M-G-*L*-Trp$_5$, M-G-*L*-Trp$_{20}$ and M-G-*L*-Trp$_{40}$ emission spectra recorded at λ_{ex} = 279 nm and 25 °C.

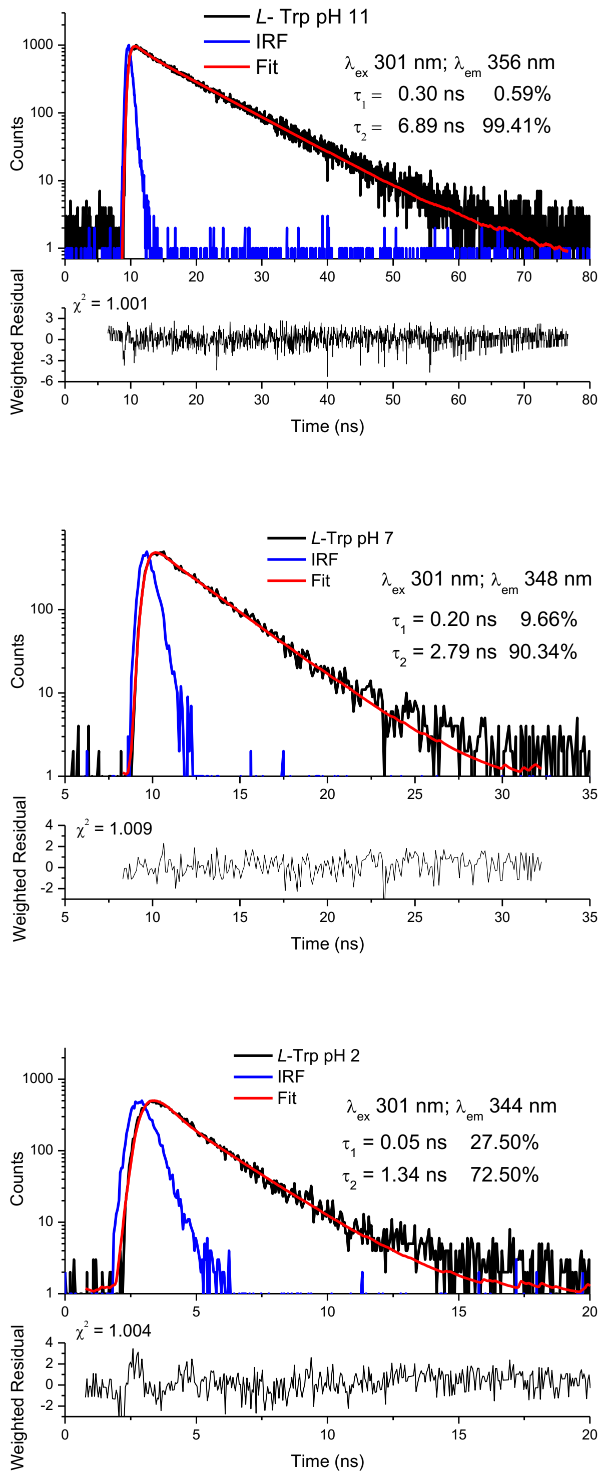

Figure S8. Emission decay of *L*-Trp vs pH at λ_{ex} = 301 nm: data (black line), instrument response function (IRF) (blue line) and convolution fit (red line). Weighted residuals are shown under the decay curves.

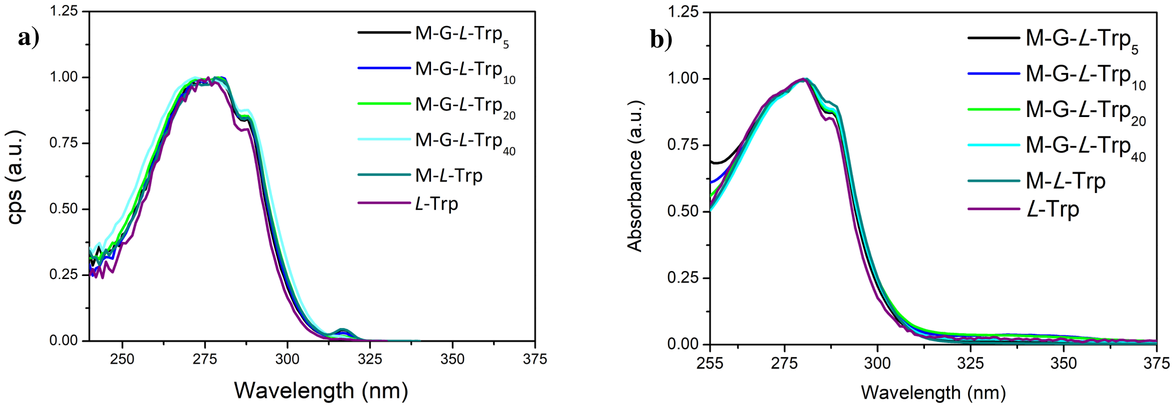

Figure S9. *L*-Tryptophan-based homo- and copolymers spectra recorded at pH 11: a) excitation (λ_{em} = 356 nm) and b) UV-vis absorption. *L*-Tryptophan is reported for comparison purposes as well.

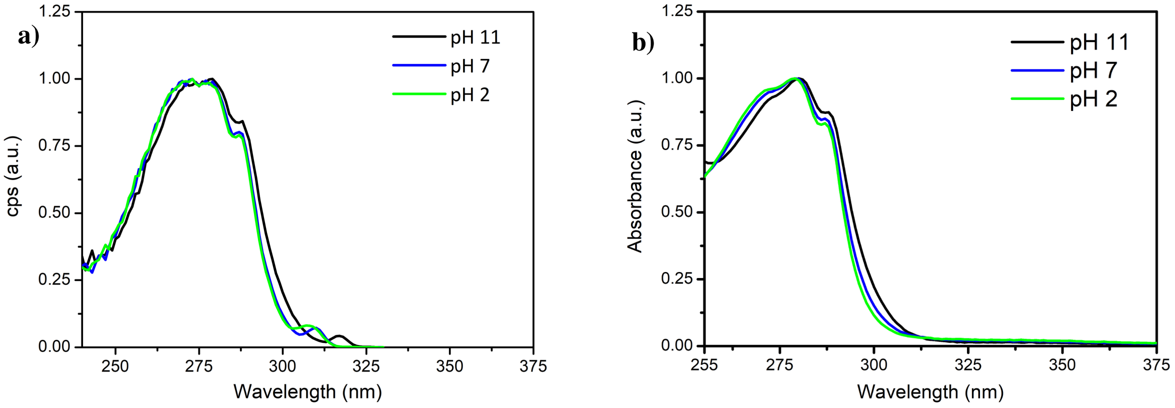

Figure S10. pH-dependence of M-G-*L*-Trp$_5$ spectra: a) excitation (λ_{em} = 356 nm) and b) UV-vis absorption.

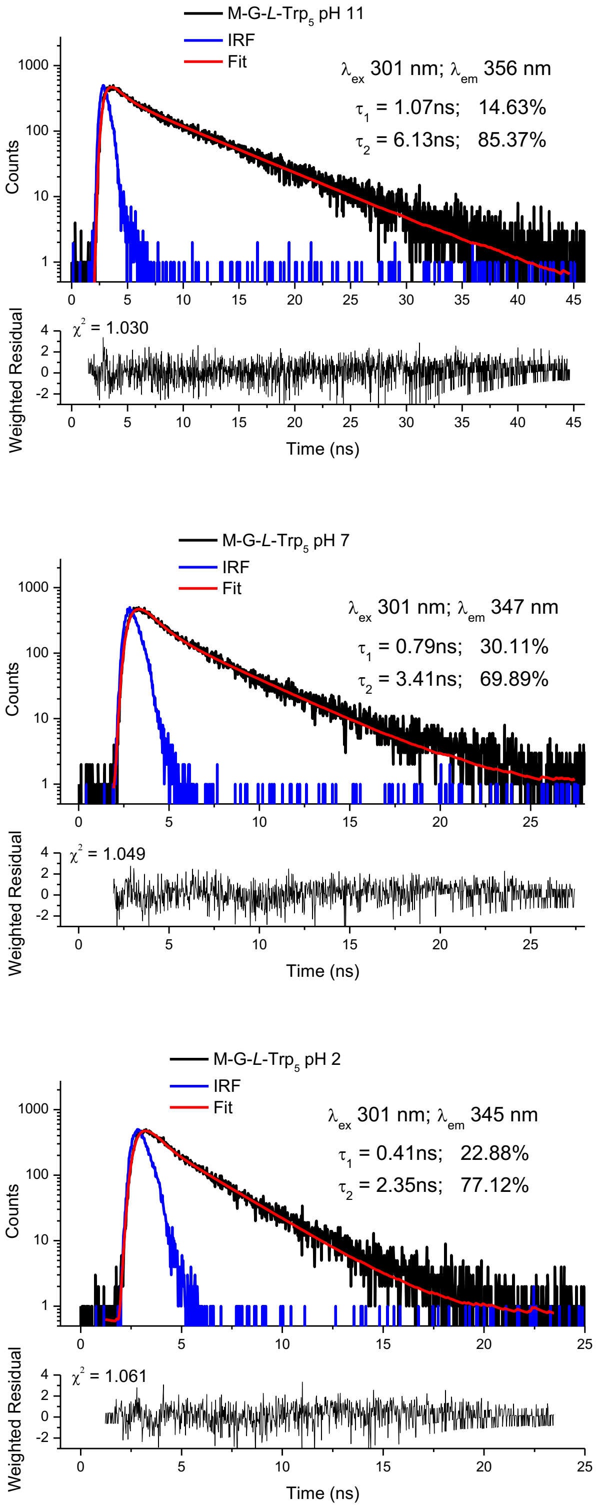

Figure S11. Emission decay of M-G-*L*-Trp_5 vs pH at λ_{ex} = 301 nm: data (black line), instrument response function (IRF) (blue line) and convolution fit (red line). Weighted residuals are shown under the decay curves.

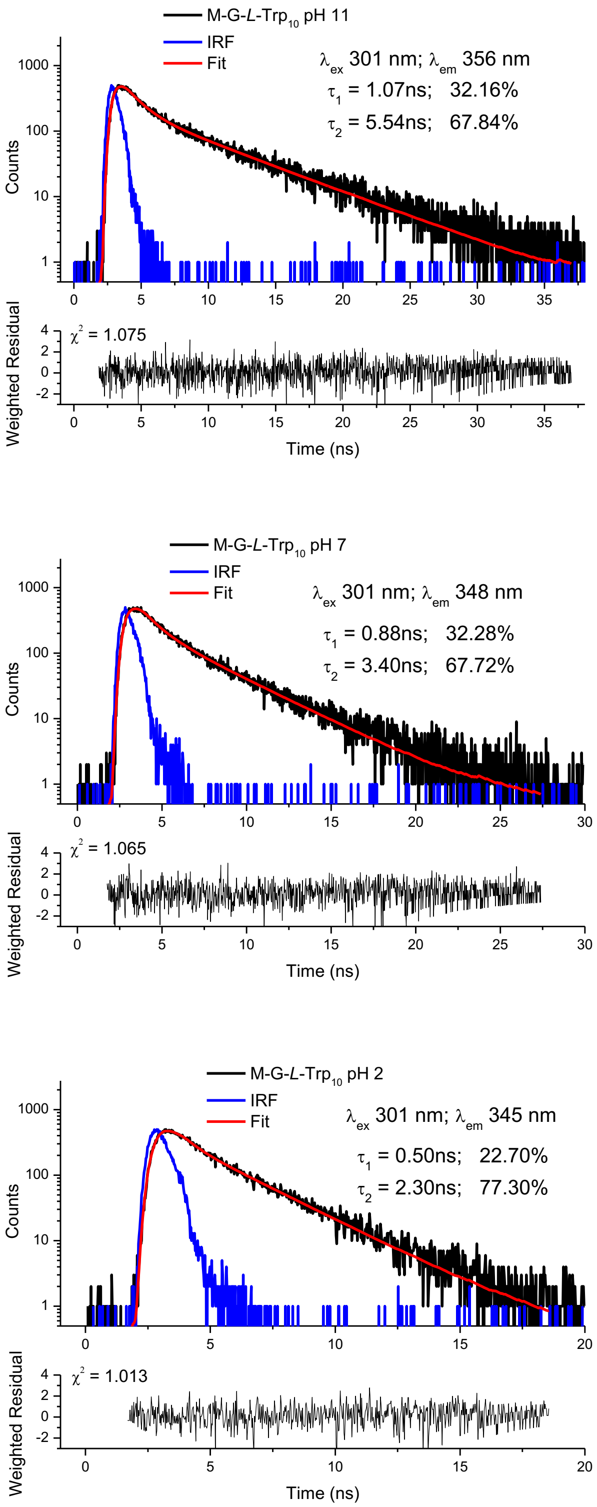

Figure S12. Emission decay of M-G-*L*-Trp_{10} vs pH at λ_{ex} = 301 nm: data (black line), instrument response function (IRF) (blue line) and convolution fit (red line). Weighted residuals are shown under the decay curves.

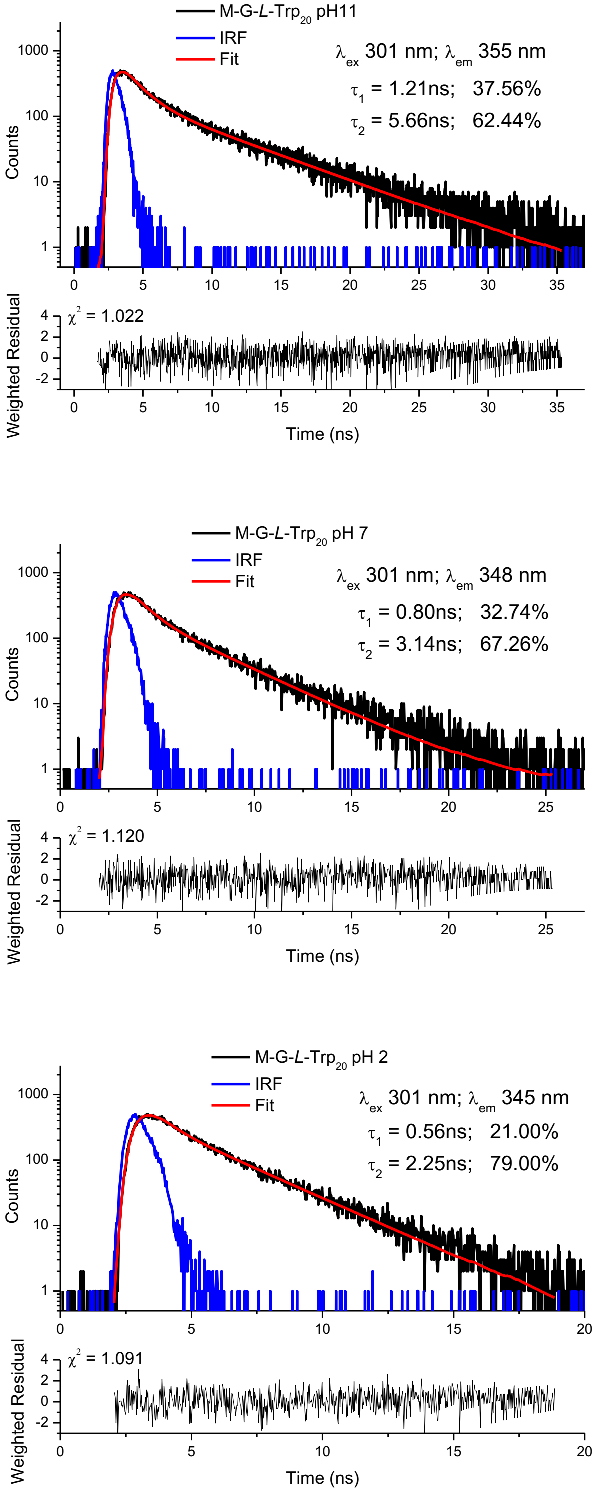

Figure S13. Emission decay of M-G-*L*-Trp_{20} vs pH at λ_{ex} = 301 nm: data (black line), instrument response function (IRF) (blue line) and convolution fit (red line). Weighted residuals are shown under the decay curves.

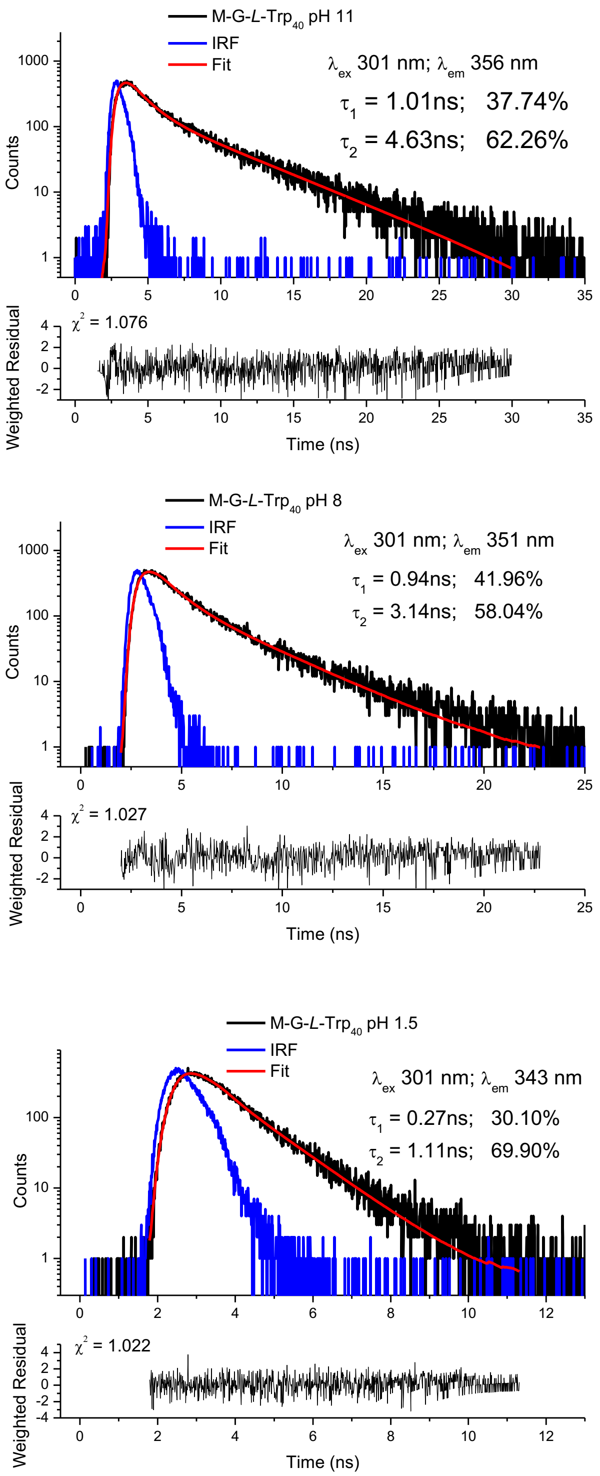

Figure S14. Emission decay of M-G-*L*-Trp40 vs pH at λ_{ex} = 301 nm: data (black line), instrument response function (IRF) (blue line) and convolution fit (red line). Weighted residuals are shown under the decay curves.

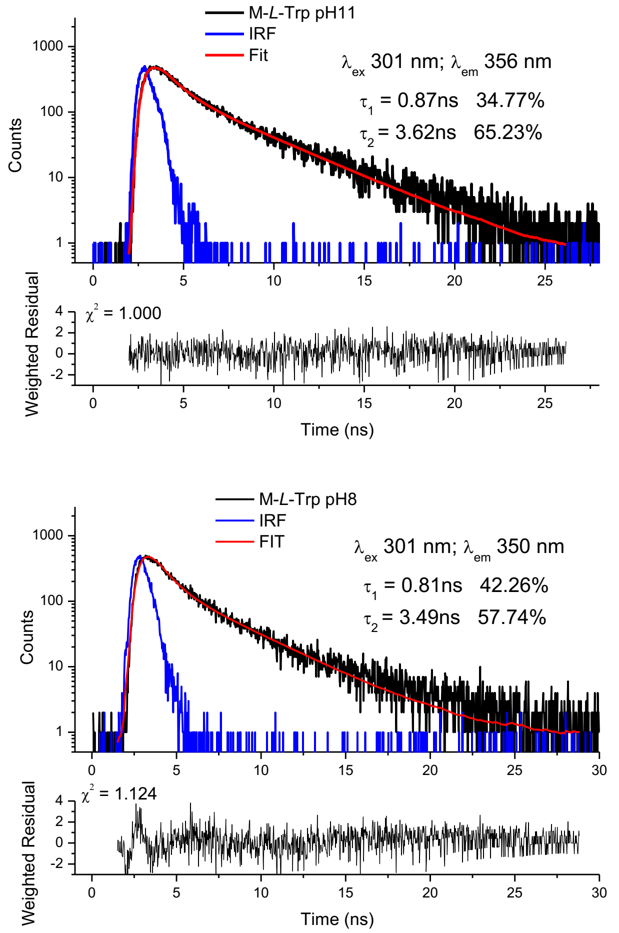

Figure S15. Emission decay of M-*L*-Trp vs pH at λ_{ex} = 301 nm: data (black line), instrument response function (IRF) (blue line) and convolution fit (red line). Weighted residuals are shown under the decay curves.

Table S1. *L*-Tryptophan-based copolymers' pK_a values, calculated from forward titration data taking into accounts solubility limits. M-G-*L*-Trp_{40} pK_a values are obtained from back-titration.

	M-G-*L*-Trp_5					
**Forward**	1st		2nd		3rd	
	-COOH	-NR_3	-COOH	-NR_3	-COOH	-NR_3
pK_a	1.96	7.68	1.95	7.74	2.22	7.92
	**M-G-*L*-Trp_{10}**					
**Forward**	1st		2nd		3rd	
	-COOH	-NR_3	-COOH	-NR_3	-COOH	-NR_3
pK_a	1.92	7.61	2.00	7.70	2.27	7.95
	**M-G-*L*-Trp_{20}**					
**Forward**	1st		2nd		3rd	
	-COOH	-NR_3	-COOH	-NR_3	-COOH	-NR_3
pK_a	1.98	7.81	1.88	7.59	2.25	7.82
	**M-G-*L*-Trp_{40}**					
**Backward**	1st		2nd		3rd	
	-COOH	-NR_3	-COOH	-NR_3	-COOH	-NR_3
pK_a	---	7.78	---	7.78	---	7.75

Table S2. Emission maximum of *L*-tryptophan, homo- and copolymers recorded by steady-state fluorescence measurements of non-degassed solutions in distilled water vs pH at λ_{ex} = 279 nm.

pH	*L*-Trp λ_{em} (nm)	M-G-*L*-Trp_5 λ_{em} (nm)	M-G-*L*-Trp_{10} λ_{em} (nm)	M-G-*L*-Trp_{20} λ_{em} (nm)	M-G-*L*-Trp_{40} λ_{em} (nm)	M-*L*-Trp λ_{em} (nm)
**11**	356	356	356	355	356	356
**7**	348	347	348	348	351[a]	350[a]
**2**	344	345	345	345	343[a]	

[a] Analyses carried out at pH 1.5-8, instead of 2 and 7, due to solubility limits.

Table S3. *L*-Tryptophan, homo- and copolymers time-resolved fluorescence measurements of non-degassed solutions in distilled water vs pH. In parentheses % of *L*-tryptophan population that decays at the calculated τ time. λ_{ex} = 301 nm.

**pH**	***L*-Trp** **τ (ns)** **(%)**	**M-G-*L*-Trp$_5$** **τ (ns)** **(%)**	**M-G-*L*-Trp$_{10}$** **τ (ns)** **(%)**	**M-G-*L*-Trp$_{20}$** **τ (ns)** **(%)**	**M-G-*L*-Trp$_{40}$** **τ (ns)** **(%)**	**M-*L*-Trp** **τ (ns)** **(%)**
**11**	τ_1 = 0.30 (0.59)	τ_1 = 1.07 (14.63)	τ_1 = 1.07 (32.16)	τ_1 = 1.21 (37.56)	τ_1 = 1.01 (37.74)	τ_1 = 0.87 (34.77)
	τ_2 = 6.89 (99.41)	τ_2 = 6.13 (85.37)	τ_2 = 5.54 (67.84)	τ_2 = 5.66 (62.44)	τ_2 = 4.63 (62.26)	τ_2 = 3.62 (65.23)
**7**	τ_1 = 0.20 (9.66)	τ_1 = 0.79 (30.11)	τ_1 = 0.88 (32.28)	τ_1 = 0.80 (32.74)	τ_1 = 0.94[a] (41.96)	τ_1 = 0.81[a] (42.26)
	τ_2 = 2.79 (90.34)	τ_2 = 3.41 (69.89)	τ_2 = 3.40 (67.72)	τ_2 = 3.14 (67.26)	τ_2 = 3.14[a] (58.04)	τ_2 = 3.49[a] (57.74)
**2**	τ_1 = 0.05 (27.50)	τ_1 = 0.41 (22.88)	τ_1 = 0.50 (22.70)	τ_1 = 0.56 (21.00)	τ_1 = 0.27[a] (30.10)	
	τ_2 = 1.34 (72.50)	τ_2 = 2.35 (77.12)	τ_2 = 2.30 (77.30)	τ_2 = 2.25 (79.00)	τ_2 = 1.11[a] (69.90)	

[a] Analyses carried out at pH 1.5-8, instead of 2 and 7, due to solubility limits.

Reference

1. De Levie, R. *How to Use ExcelW in Analytical Chemistry and in General Scientific Data Analysis*; Cambridge University Press: Cambridge, **2001**.
